# Integrated metabolome and microbiome analysis deciphers the effects of resveratrol and β-hydroxy-β-methylbutyric acid on jejunal function under different protein levels in Tibetan sheep

**DOI:** 10.1128/spectrum.02843-25

**Published:** 2026-01-21

**Authors:** Kaina Zhu, Yu Zhang, Fengshuo Zhang, Qiurong Ji, Quyangangmao Su, Zhenling Wu, Xuan Chen, Tingli He, Zhiyou Wang, Shengzhen Hou, Linsheng Gui

**Affiliations:** 1 College of Agriculture and Animal Husbandry, Qinghai University 207475, Xining, Qinghai, China; Nanchang University, Nanchang, Jiangxi, China

**Keywords:** resveratrol, β-hydroxy-β-methylbutyric acid, jejunum morphology, microbiota, metabolomics, Tibetan sheep

## Abstract

Research has demonstrated that resveratrol (RES) and β-hydroxy-β-methylbutyric acid (HMB) promote gastrointestinal health by altering the intestinal microbiome. The present study aimed to evaluate the impacts of RES and HMB on the bacterial community composition and metabolomic profiles of the jejunum in Tibetan sheep. A total of 120 male Tibetan lambs, with an average initial body weight of 16.87 ± 0.31 kg, were randomly assigned to four experimental groups according to a 2×2 factorial design. The experimental diets contained 11.19% protein diet, or 12.69% protein diet supplemented with RES/HMB (H-RES-HMB and L-RES-HMB, respectively), and 11.19% protein diet, or 12.69% protein diet without supplemented with RES/HMB (LCP and HCP, respectively). The findings indicated a significant increase in jejunal digestive enzyme activities (α-amylase and chymotrypsin), antioxidant capacity (catalase, glutathione peroxidase, and superoxide dismutase), and immune responses (including immunoglobulins A, G, and M) in the H-RES-HMB group, in contrast to a notable decrease in markers of oxidative stress and pro-inflammatory cytokines (interleukin 1β, interleukin 6, and tumor necrosis factor alpha) (*P* < 0.05). Morphological assessments revealed a significant reduction in crypt depth within the H-RES-HMB group (*P* < 0.05). Among the short-chain fatty acids, butyric acid concentration was significantly elevated in the H-RES-HMB group (*P* < 0.05). Additionally, mRNA expression levels of barrier-associated genes, including *OCLN, Muc-2,* and *ZO-1* in the jejunum, were significantly enhanced in the H-RES-HMB group. Microbial community analysis demonstrated that the inclusion of RES and HMB in the 14% protein diet resulted in an increase in the relative abundance of *Firmicutes*, *Bacillus*, and *Methanobrevibacter*, while simultaneously reducing the abundance of *Proteobacteria, Escherichia,* and *Shigella*. Notably, the increased abundance of these beneficial bacteria was positively correlated with butyric acid concentrations. Furthermore, metabolomic analysis indicated higher levels of L-Arginine and His-Lys in the H-RES-HMB group, which also showed a positive correlation with butyric acid concentration, whereas isovaleric acid, D-xylose, and diacetyl concentrations were lower in this group. In conclusion, the findings suggest that a 14% protein diet is more effective in enhancing jejunal morphology and barrier function. Supplementation with RES and HMB in this diet resulted in elevated concentrations of volatile fatty acids, particularly butyric acid, by modulating microbial community composition (notably *Firmicutes, Bacillus,* and *Methanobrevibacter*) and metabolite profiles (including Gamma-aminobutyric acid, Succinate, L-Arginine, and His-Lys). A synergistic effect was found between the crude protein level (14%) and dietary RES/HMB on villus phenotype, digestive enzyme capacity, and antioxidant activity.

**IMPORTANCE:**

To thrive in harsh, high-altitude environments, Tibetan sheep require efficient nutrient absorption. Our study shows that a diet with optimal protein (12.69%) supplemented with natural additives—resveratrol and β-hydroxy-β-methylbutyric acid—significantly enhances gut health. This dietary regimen strengthened the intestinal barrier, suppressed harmful inflammation, and boosted local immunity. Furthermore, it enriched the gut microbiome with beneficial butyric acid-producing bacteria, a key nutrient for intestinal cells. This research establishes a practical nutritional strategy to improve intestinal function and overall resilience in Tibetan sheep, supporting sustainable livestock production in challenging plateau regions.

## INTRODUCTION

The gut ecosystem in ruminants harbor complex microbial communities, which play a crucial role in host nutrient metabolism, immune regulation, and health (1). Growing researchers have demonstrated a close correlation between the gastrointestinal microbes and the efficiency of nutrient metabolism, growth performance, and health status of the host. On the Qinghai–Tibet plateau, cold season feeding strategies drove plastic adaptation of rumen function in Tibetan sheep by regulating microbial community structure and metabolic profiles (2). The dominant genera of rumen microbiota were influenced by seasonal diet factors rather than genetics in Tibetan sheep (3). Previous studies have demonstrated that the optimal dietary protein level (11.58%) increased the abundance of *Romboutsia* and *Bifidobacterium* in the jejunum, which were positively associated with improved meat tenderness and water-holding capacity of Tibetan sheep (4). When fed a low-protein diet, the dietary lysine/methionine ratio influenced the concentration of the jejunal short-chain fatty acids (SCFAs) by modulating the microbial community (5).

Resveratrol (RES) is a natural polyphenol found in many fruits and vegetables, such as grapes and peanuts (6, 7). Polyphenols and their metabolites are effective in treating various inflammatory diseases and cancers of the gastrointestinal tract (8). At present, the anti-inflammatory, anti-cancer, anti-oxidant, anti-diabetic, and other functions of RES are well-known. It is currently also a popular dietary supplement. Studies have shown that RES supplementation can improve intestinal health in suckling pigs by altering the flora and reducing inflammation in the intestine (7, 9). A study investigating the impact of heat stress on broiler chickens found that dietary supplementation with RES altered the composition of the gut microbiota, influenced the expression of intestinal tight junction mRNA, and improved intestinal function (10). The β-hydroxy-β-methylbutyric acid (HMB), a popular supplement, is a metabolite of leucine, primarily found in protein-based foods (11). Dietary supplementation of HMB not only improved intestinal morphology, but also improved intestinal function (12). Supplementation of HMB in pregnant sows can also improve piglet health and growth (13). Studies have shown that HMB plays a crucial role in antioxidant capacity and immune status (14). Research has found that supplementing the diet with RES and HMB enhances the intestinal health of Tibetan sheep by regulating the abundance of intestinal microbiota and metabolites, thereby increasing SCFA concentrations (15). Furthermore, studies have confirmed that RES and HMB supplements improve the gut health by increasing the digestive enzyme activity in the duodenum and enhancing the antioxidant capacity and immune function (16). These results suggest that RES and HMB are essential for maintaining the intestinal health of Tibetan sheep.

Currently, few studies have reported the effects of protein levels and supplements of RES and HMB on the jejunal function of Tibetan sheep. Based on the biological activities of these two substances, we speculate that protein levels and supplements can effectively improve the intestinal health of Tibetan sheep. To this end, this study systematically explored the effects of dietary protein levels, RES, and HMB on jejunal histomorphology, immune and antioxidant capacities, digestive enzyme activities, lipopolysaccharide content, and the jejunal microbiota and metabolites of Tibetan sheep.

## MATERIALS AND METHODS

### Animal management and experimental design

A total of 120 60-day-old male lambs with an initial mean body weight of 16.87 ± 0.31 kg (mean±standard deviation) were selected and randomly allocated into four treatments, following 2×2 factorial design. Each treatment included five replicates (*n* = 5), with six sheep housed per pen. For the experimental treatments, the lambs were fed the diets with 11.19% crude protein (CP) level non-supplemented (L), 11.19% protein with RES and HMB (L-RES-HMB), 12.69% CP level non-supplemented (H), and 12.69% protein with RES and HMB (H-RES-HMB). The primary variables examined were two dietary levels of CP at either 11.19% or 12.69%, along with the absence or presence of RES (1.50 g/day) and HMB (1.25 g/day). During the preparation of the experimental feeds, both RES and HMB were firstly supplemented to the premix, and then mixed with the concentration in the blender. The fattening program lasted for 100 days, with 10 days of pre-trial period and 90 days of trial period. Feed composition and nutrient levels were provided in Table 1.

**TABLE 1:** Composition of the basic diet (% dry matter basis)

Items	L-CP	H-CP
Oat hay	15	15
Corn silage	15	15
Corn	40.81	36.05
Soybean meal	0.7	1.4
Rapeseed meal	4.9	8.96
Cottonseed meal	1.4	1.4
Palm meal	17.5	17.5
NaCl	0.7	0.7
Limestone	0.7	0.7
NaHCO_3_	0.07	0.07
Premix^*a*^	3.22	3.22
Total	100	100
Nutrient levels^*b*^		
Digestible energy (MJ·kg^−1^)	11.77	11.68
CP (%)	11.19	12.69
Ether extract (%)	3.24	3.13
Crude fiber (%)	15.24	15.65
Neutral detergent fiber (%)	33.23	33.69
Acid detergent fiber (%)	22.83	23.43
Ca (%)	0.65	0.69
*P* (%)	0.35	0.35

^*a*^ Premixes provide Cu 18 mg, Fe 66 mg, Zn 30 mg, Mn 48 mg, Se 0.36 mg, I 0.6 mg, Co 0.24 mg, VA 24000 IU, VD 4 800 IU, and VE 48 IU per kg of feed.

^*b*^ Digestible energy is calculated and the rest is measured.

### Sample collection and processing

At the end of the trial, after fasting for 24 h, one sheep from each replicate was randomly selected (*n* = 6 per treatment). All selected sheep were stunned and slaughtered at a commercial slaughterhouse. Approximately 25 mg of the jejunal digesta were sampled and stored in dry ice immediately for further analysis. From the middle section (4 cm) of the jejunum, approximately 3×3 cm tissue sample was taken, rinsed with normal saline, and fixed in 4% paraformaldehyde solution for 24 h for histomorphology analysis. The jejunum tissue was excised and stored in 50 mL sterile enzyme-free frozen storage tube for enzyme-linked immunosorbent assay (ELISA) and jejunum barrier tests.

### Measurement of antioxidant, immunity, and digestive enzymes in the jejunum

The jejunal digesta were centrifuged at 3,000 rpm for 20 min at 2–8°C and the supernatant was collected. This preparation was then centrifuged again at 3,000 rpm for 20 min at the same temperature range, and the supernatant was collected once more. Subsequently, ELISA tests were conducted to assess various antioxidant markers, including catalase (CAT), glutathione peroxidase (GSH-PX), superoxide dismutase (SOD), total antioxidant capacity (T-AOC), and malondialdehyde (MDA). Furthermore, immune markers such as immunoglobulins A, G, and M (IgA, IgG, and IgM), interleukin 1β (IL-1β), interleukin 6 (IL-6), and tumor necrosis factor alpha (TNF-α) in jejunal tissues were evaluated. In addition, the concentrations of digestive enzymes, namely cellulase, lipase, α-amylase, trypsin, and chymotrypsin, present in the jejunal contents were quantified (Enzyme Immune Industrial Co., Ltd., Jiangsu, China) and analyzed at a wavelength of 450 nm using an enzyme label apparatus.

### Morphological analysis of jejunal tissue

Sections were examined by hematoxylin and eosin (HE) staining to measure jejunal villus height (VH), villus width (VW), crypt depth (CD), VH/CD ratio, thickness of mucosa (TM), and thickness of muscularis (MT). Briefly, the fixed jejunal tissue was dehydrated using ethanol, soaked in xylene, and embedded in paraffin blocks. Then, 3 µm thick sections of cooled wax blocks were prepared and placed on a slide. Paraffin sections were dewaxed using xylene and anhydrous ethanol gradient and rinsed with distilled water. Then HE staining was performed. The paraffin was dehydrated again, and the slices were sealed with neutral gum and examined immediately. Finally, five well-orientated sections of the villi and its accompanying crypts of each sample were prepared using an optical microscope (DP26, OLYMPUS). Images were analyzed using CaseViewer software (version 2.4).

### Quantitative polymerase chain reaction (qPCR)

Total RNA was isolated from the jejunal tissue sample utilizing TRIzol reagent. The quantification and assessment of RNA quality were performed using a NanoDrop 2000 spectrophotometer. Subsequently, the isolated total RNA was utilized to synthesize complementary DNA (cDNA) using the Universal SYBR Green qPCR Mix Kit (Azaood, Beijing, China). The qPCR reaction mixture included 10 μL of 2× SYBR Green Pro Taq HS premix, 0.4 μL each of forward and reverse primers, 2 μL of cDNA, and 7.2 μL of nuclease-free water. The thermal cycling conditions for the PCR were set to 30 at 95°C, followed by 40 cycles consisting of 10 s at 95°C and 30 s at 60°C. The relative gene expression levels were determined using the 2^−△△Ct^ calculation method. The primer sequences are detailed in Table S1.

### Determination of SCFAs

SCFAs content, including acetic acid, propionic acid, butyric acid, valeric acid, and isovaleric acid, was determined in the jejunum. The sample was extracted following the method of Ji et al. (17), and the content of SCFAs was detected by gas chromatography. The SCFAs content was detected by gas chromatography (7890B GC System) equipped with a DB-FFAP capillary column (30 m×250 μm×0.25 μm). The rate of increase in program temperature was: initial temperature of 90°C; temperature rise to 160°C at the rate of 10 °C/min; then the increase in temperature to 240°C at 40°C/min and maintained for 5 min.

### DNA extraction and 16S rDNA sequencing

The microbial DNA of jejunum contents was extracted using HiPure Stool DNA Kits (Magen, Guangzhou, China). DNA concentration and purity were determined by NanoDrop 2000 microspectrophotometer. DNA quality was detected on 1% agarose gel. The specific primers 341F (5′-CCTACGGGGNGGCWGCAG-3′) and 806R (5′-GGACTACHVGGGTATCTAAT-3′) with barcode were used to amplify the V3-V4 region of the 16S rRNA gene. PCR amplification is the same as that used by Ji et al. (17). Sequencing libraries were constructed using the Illumina DNA Prep Kit (Illumina, CA, USA), with the quality of the libraries evaluated utilizing the ABI StepOnePlus Real-Time PCR System (Life Technologies, Foster City, USA). Subsequently, the sequencing of the libraries was conducted using the PE250 mode pooling on the Novaseq 6000 platform (NovaSeq6000 S2 Reagent Kit v1.5, Illumina, USA). The resulting raw data were deposited in the NCBI Sequence Read Archive (SRA) database.

Raw data were filtered using FASTP (0.18.0) (18) and merged using FLASH (1.2.11) (19). Next, UPARSE (9.2.64) (20) was used to cluster the operational taxonomic units (OTUs) with a similarity of ≥97%, and then UCHIME (21) algorithm was used to identify and remove chimeras. The RDP (2.2) (22) annotation software was used to annotate the OTU representative sequence SILVA (138.1) (23) database for species classification, with a confidence threshold set at 0.8.

### Metabolite extraction and analysis

The samples were defrosted at a temperature of 4°C, and a suitable volume of the samples was combined with a pre-chilled solution of methanol, acetonitrile, and water (in a ratio of 2:2:1, vol/vol/vol) for treatment. The resulting mixture underwent vortex mixing and was subjected to low-temperature ultrasound treatment for a duration of 30 min. Following this, the mixture was allowed to rest at −20°C for 10 min, facilitating protein precipitation. The supernatant was then collected and concentrated using a vacuum centrifuge. For subsequent mass spectrometry analysis, 100 μL of an acetonitrile solution (acetonitrile: water = 1:1, vol/vol) was added to the sample, ensuring thorough dissolution and mixing via vortexing. This solution was centrifuged at 14,000 rpm at 4°C for 15 min, with the supernatant being retained for analysis. Additionally, a quality control sample was incorporated into the sample sequence to assess system stability and data reliability.

Analysis was conducted utilizing ultra-high performance liquid chromatography (1290 Infinity LC, Agilent) in conjunction with a quadrupole time-of-flight mass spectrometer (AB Sciex TripleTOF 6600). For the purpose of separation via hydrophilic interaction liquid chromatography, an ACQUIY UPLC BEH column (2.1 mm × 100 mm, 1.7 µm; Waters, Ireland) was used. The Collection of Algorithms for MEtabolite pRofile Annotation package facilitated the annotation of the detected peaks, while the MS2 database was utilized for the identification of metabolites.

Data from ESI+ and ESI- models were analyzed through principal component analysis using R package (24) gmodels. Metabolic differences between groups were determined using partial least squares discriminant analysis (PLS-DA) using the R language package ropls (25). Metabolites with VIP > 1, *P* < 0.05, and Log_2_|FC| > 1 were considered significantly different. Finally, the differential metabolites (DMs) were analyzed through Kyoto Encyclopedia of Genes and Genomes for metabolite pathway enrichment and trend analysis.

### Statistical analysis

Pearson’s correlation test was employed to analyze the correlation between jejunal antioxidants, immunity, digestive enzymes, gene expression related to barrier function, jejunal microbiota, and metabolites related to short-chain fatty acids. Data are expressed as means ± standard error of the mean, with *P* < 0.05 indicating significant difference. Metabolomic statistics were analyzed using ProteoWizard to convert raw MS data into MzXML format and subsequently imported into XCMS software. Pearson’s correlation test was used to analyze the correlation between jejunal tissue antioxidants and immunity, jejunal digestive enzymes and lipopolysaccharides, and the expression of genes associated with barrier function and metabolites related to jejunal flora. Graphs were generated using the R language package Psych (1.8.4) (26) and Origin 2021.

## RESULTS

### Digestive enzyme activity of jejunum contents of Tibetan sheep

**Fig 1:**
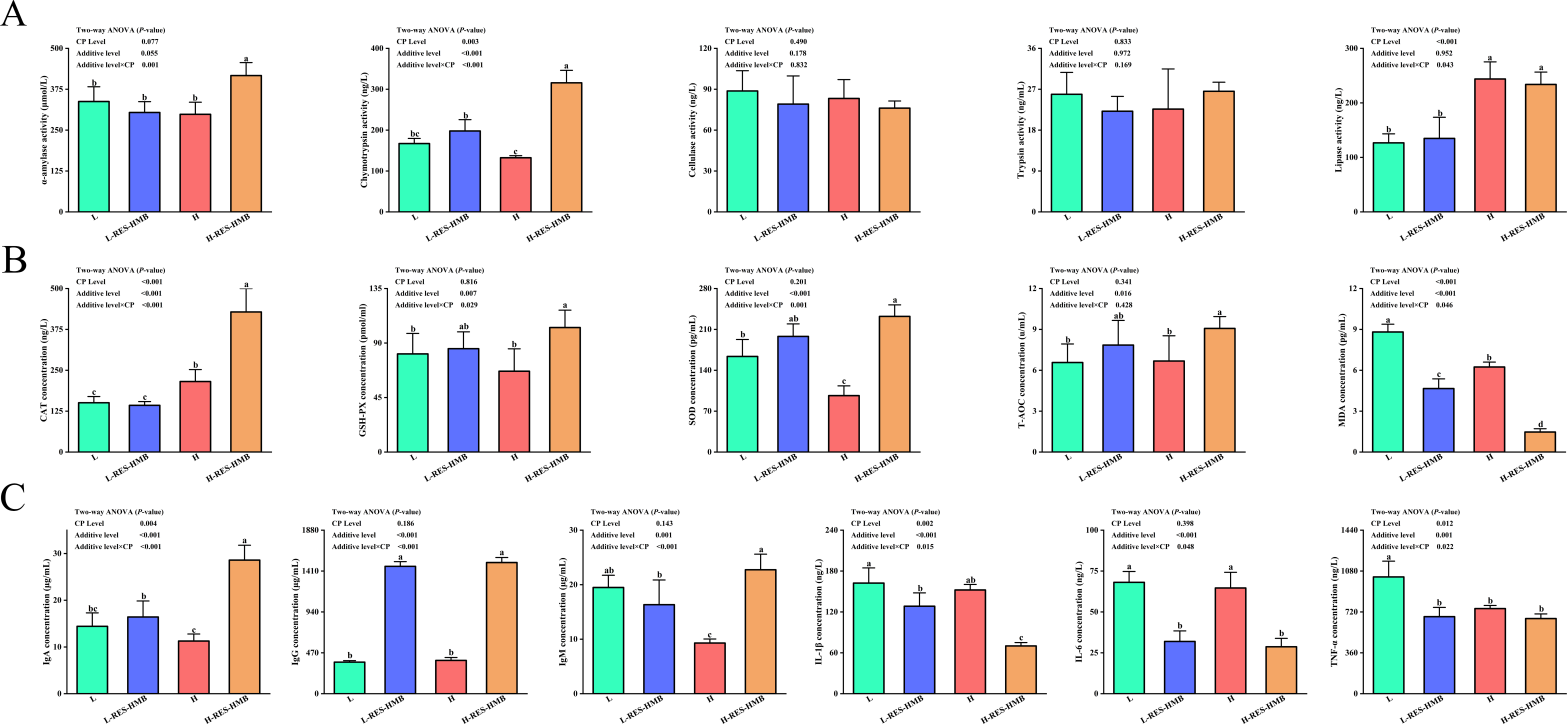
(**A**) Effects of different protein diets supplemented with RES and HMB on jejunal digestive enzyme activities. (**B**) Effects of different protein diets supplemented with RES and HMB on the antioxidant capacity of jejunum. (**C**) Effects of different protein diets supplemented with RES and HMB on jejunum immune response. “CP Level” (12% protein and 14% protein), “RES-HMB” (non-supplemented; or HMB: 1.25g /day, RES: 1.50 g/day). “Additive × CP Level” indicates the interaction of RES and HMB with dietary protein level. The figure marked with different lowercase letters indicates significant difference (*P* < 0.05), while the figure marked without lowercase letters indicates no significant difference (*P* > 0.05).

The effects of the treatments on digestive enzyme activities of the jejunal contents are presented in Fig. 1A. The diet with 14% protein significantly increased chymotrypsin and lipase levels (*P* < 0.05). Further, the addition of RES and HMB significantly increased chymotrypsin levels (*P* < 0.05). An interaction effect between levels of CP level and RES and HMB supplementation was observed for α-amylase, chymotrypsin, and lipase (*P* < 0.05). The 14% CP diet supplemented with RES and HMB showed a synergistic effect, and the α-amylase and chymotrypsin levels were significantly higher in the H-RES-HMB group (*P* < 0.05).

### Antioxidant capacity of jejunum tissue of Tibetan sheep

The impact of the experimental treatments on the antioxidant capacity of jejunal tissue is illustrated in Fig. 1B. The diet containing 14% protein resulted in a notable increase in CAT levels (*P* < 0.05) alongside a significant decrease in MDA levels (*P* < 0.05). The incorporation of RES and HMB led to a marked enhancement in the levels of CAT, GSH-PX, SOD, and T-AOC (*P* < 0.05), while concurrently decreasing MDA levels (*P* < 0.05). Moreover, an interaction effect was identified between the CP levels and the supplementation of RES and HMB on the levels of CAT, GSH-PX, SOD, and MDA (*P* < 0.05). Additionally, the H-RES-HMB group exhibited elevated levels of CAT, GSH-PX, and SOD compared to the L, L-RES-HMB, and H groups, with a significantly lower MDA content observed in the H-RES-HMB group (*P* < 0.05).

### Immune response of jejunum tissue in Tibetan sheep

The effects of the experimental treatments on the immunological activity of the jejunal tissue are presented in Fig. 1C. Feeding the 14% CP diet significantly reduced TNF-α levels (*P* < 0.05). The incorporation of RES and HMB led to a marked enhancement in the levels of IgA and IgM (*P* < 0.05), while concurrently decreasing IL-1β levels (*P* < 0.05). An interaction effect was identified between the CP levels and the supplementation of RES and HMB on the levels of IgA, IgG, IgM, IL-1β, IL-6, and TNF-α (*P* < 0.05). Additionally, the H-RES-HMB group significantly elevated levels of IgA compared to the L, L-RES-HMB, and H groups, with a significantly lower IL-1β content observed in the H-RES-HMB group (*P* < 0.05).

### Histomorphological analysis

**Fig 2:**
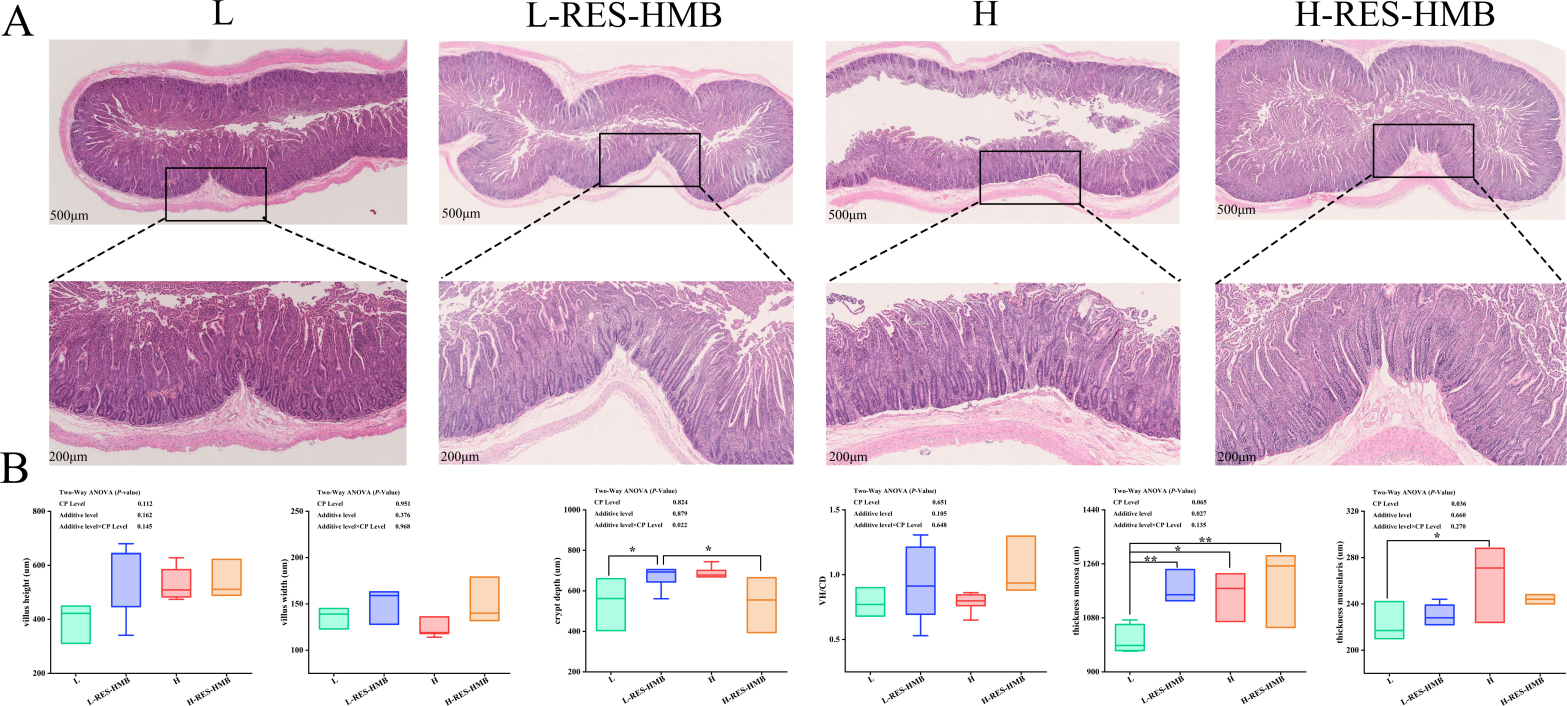
Effects of different protein diets supplemented with RES and HMB on jejunum morphology. (**A**) Observation of jejunum morphology (hematoxylin-eosin staining, 500× and 200× μm). (**B**) Boxplot analysis of jejunum tissue morphological parameters, “*” *P* < 0.05, “**” *P* < 0.01.

The histomorphological observation of the jejunum is shown in Fig. 2A. HE staining of the jejunum showed that the CP level significantly affected the jejunal MT (*P* < 0.05). The additive level had a significant effect on the jejunal MT mucosa. An interaction effect between CP level and RES and HMB supplementation was noted on CD (*P* < 0.05), and the CD levels were lower in the H-RES-HMB group than in L, L-RES-HMB, and H groups (Fig. 2B).

### Expression of jejunum barrier-related genes in Tibetan sheep

**Fig 3:**
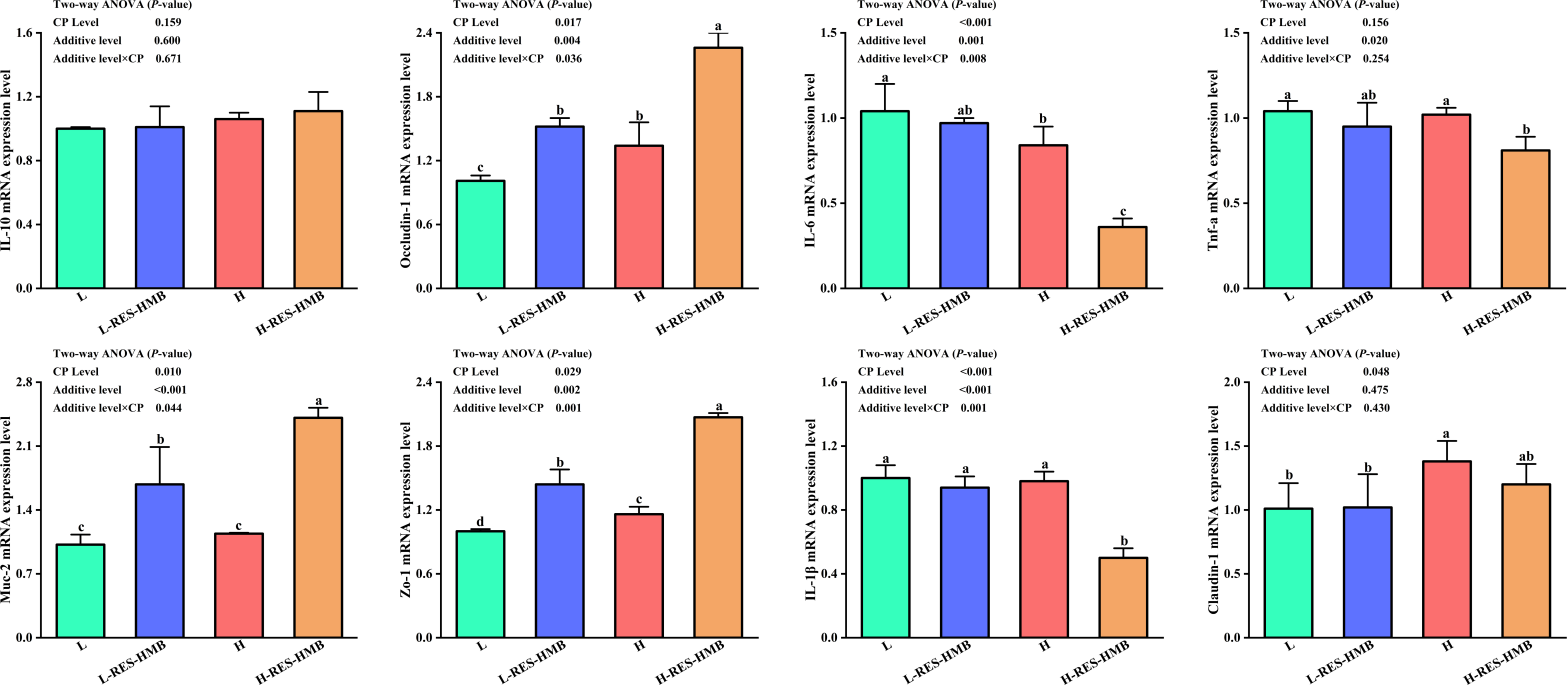
Expression of jejunum barrier-related genes. Marked with different lowercase letters indicates significant difference (*P* < 0.05), while marked without lowercase letters indicates no significant difference (*P* > 0.05).

Expression of related genes in jejunal tissues is presented in Fig. 3. Feeding a 14% CP diet significantly enhanced the mRNA expression of *OCLN*, *mucin-2 (Muc-2*), *zonula occludens 1 (ZO-1*), and *claudin-1*, while decreased that of IL-6 and IL-1β (*P* < 0.05). Meanwhile, the addition of RES and HMB significantly enhanced the mRNA expression of *OCLN, Muc-2, Zo-1*, and decreased that of IL-6, TNF-α, and IL-1β (*P* < 0.05). Likewise, the addition of RES and HMB in the 14% protein diet significantly increased the mRNA expression of *OCLN*, *Muc-2*, and *ZO-1*, but decreased that of IL-6 and IL-1β (*P* < 0.05).

### SCFA content

The effects of the experimental treatments on the SCFA content of the jejunum are shown in Table 2. Feeding a 14% CP diet significantly enhanced butyric acid content (*P* < 0.05). Likewise, the addition of RES and HMB significantly enhanced butyric acid (*P* < 0.05). An interaction effect between CP level with RES and HMB supplementation was noted for butyric acid (*P* < 0.05), and the butyric acid content was significantly higher in the H-RES-HMB group (*P* < 0.05).

**TABLE 2:** Effects of RES and HMB supplementation at different protein levels on SCFAs (%) of jejunum contents in Tibetan sheep

		Acetic acid	Propanoic acid	Butyric acid	Valeric acid	Hexanoic acid	Isovaleric acid
Groups	L	86.88 ± 1.97^a*d*^	5.79 ± 1.68	1.69 ± 0.33^d^	0.82 ± 0.26	2.39 ± 0.57	2.42 ± 0.38^ab^
	L-RES-HMB	85.21 ± 0.74^a^	5.85 ± 0.66	4.38 ± 0.14^b^	1.20 ± 0.33	1.73 ± 0.49	1.60 ± 0.51^bc^
	H	87.12 ± 0.42^a^	4.76 ± 0.62	2.53 ± 0.18^c^	1.03 ± 0.07	2.10 ± 0.05	3.38 ± 0.85^a^
	H-RES-HMB	84.33 ± 1.90^b^	5.07 ± 1.42	7.20 ± 0.59^a^	0.79 ± 0.40	1.72 ± 0.34	1.12 ± 0.35^c^
CP Level^*a*^	LCP	86.04 ± 1.61	5.82 ± 1.14	3.04 ± 1.49^b^	1.01 ± 0.0.34	2.06 ± 0.60	2.01 ± 0.60
	HCP	85.72 ± 1.96	4.92 ± 0.99	4.86 ± 2.59^a^	0.91 ± 0.29	1.91 ± 0.30	1.25 ± 1.37
Additive level^*b*^	N-RES-HMB	87.00 ± 1.28^a^	5.28 ± 1.27	2.11 ± 0.51^b^	0.93 ± 0.20	2.25 ± 0.39	2.90 ± 0.79^a^
	RES+HMB	84.77 ± 1.38^b^	5.46 ± 1.08	5.79 ± 1.59^a^	0.96 ± 0.0.30	1.73 ± 0.38	1.36 ± 0.47^b^
*P*-value	CP Level	0.708	0.224	<0.001	0.576	0.547	0.479
	Additive level	0.027	0.794	<0.001	0.700	0.061	0.001
	Additive level×CP Level^*c*^	0.512	0.857	0.001	0.105	0.566	0.054

^*a*^ Indicates dietary protein level (11.19% protein and 12.69% protein).

^*b*^ Indicates no supplementation and RES (1.5 g/day) and HMB (1.25 g/day).

^*c*^ Indicates the interaction of RES and HMB with dietary CP level.

^*d*^ Data in the same column with the same or no lowercase letters (a, b, c, d) indicate nonsignificant differences (*P* > 0.05) and data with different lowercase letters (a, b, c, d) indicate significant differences (*P* < 0.05).

### Composition of jejunum bacterial community

#### Diversity of microbial communities in the jejunum of Tibetan sheep

**Fig 4:**
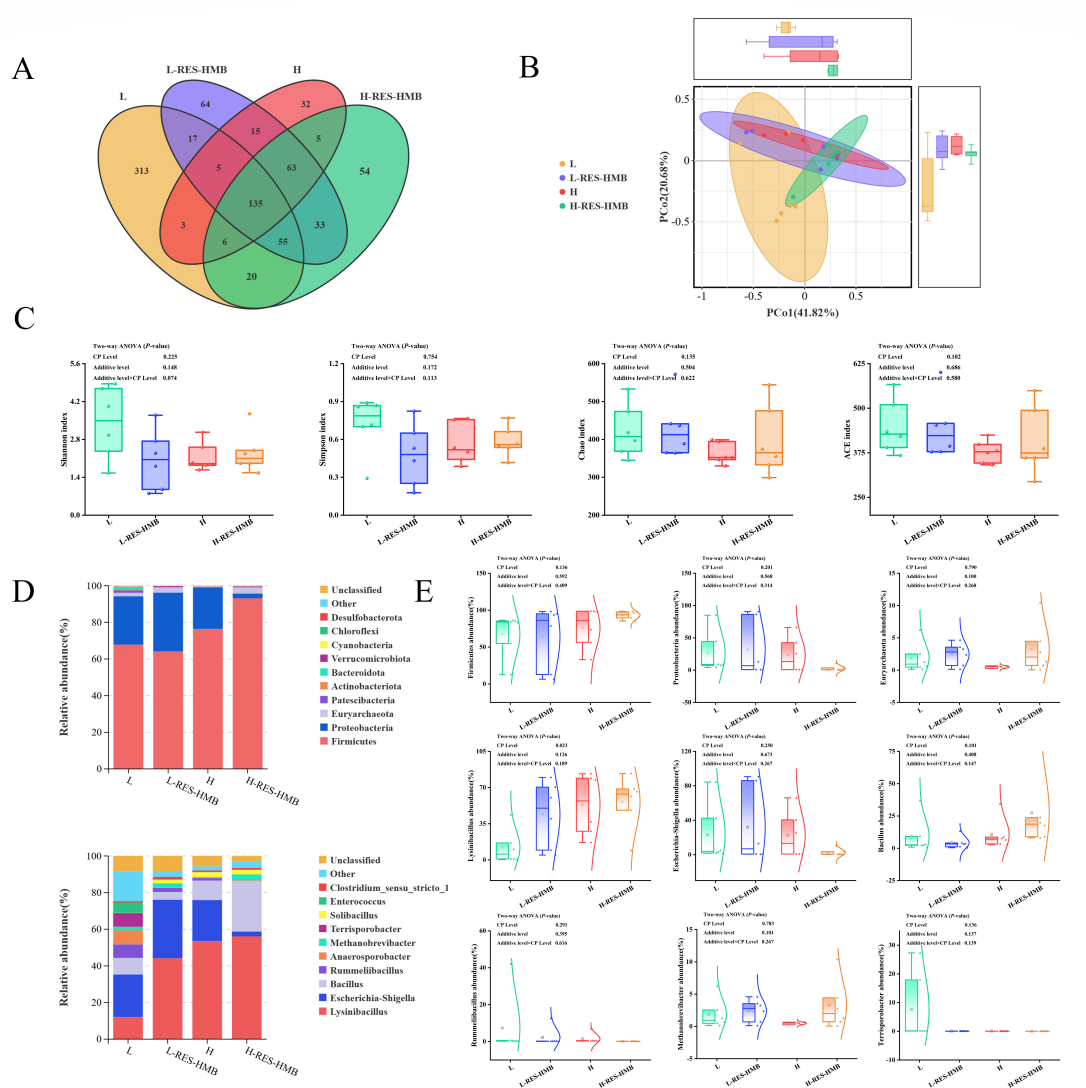
Microbial composition of jejunum. (**A**) Venn diagram analysis. (**B**) PCoA and Adonis test. (**C**) Alpha diversity analysis. (**D**) Relative abundance of flora at phyla and genus levels. (**E**) Box plot + normal distribution map shows the distribution of flora at phylum and genus level in L, L-RES-HMB, H, and H-RES-HMB groups.

16S rDNA of 24 jejunum content samples was sequenced. The Venn diagram shows a total of 820 OTUs, among which the L, L-RES-HMB, H, and H-RES-HMB had 313, 64, 32, and 54 OTUs, respectively, and the four groups shared 135 OTUs (Fig. 4A). The beta diversity was shown in terms of principal coordinate analysis (PCoA) according to Bray-Curtis distance (Fig. 4B); the four treatments never exhibited obvious separation. The alpha diversity analysis Chao1, ACE, Shannon, and Simpson indices (Fig. 4C) showed no significant difference among these indices (*P* > 0.05), indicating that the number of bacteria in each group was quite similar.

#### Difference in jejunum microbial composition in Tibetan sheep

Figure 4D shows the top 10 bacterial groups at phylum and genus levels. *Firmicutes*, *Proteobacteria,* and *Euryarchaeota* are the top three dominant bacteria groups. The relative abundance of *Firmicutes* (93.09%) and *Euryarchaeota* (3.29%) increased in the H-RES-HMB group, while that of *Proteobacteria* (2.63%) decreased in the H-RES-HMB group. At the genus level, *Lysinibacillus*, *Escherichia-Shigella*, and *Bacillus* were the top three dominant groups. Further, the relative abundance of *Lysinibacillus* (60.05%), *Bacillus* (29.41%), and *Methanobrevibacter* (3.48%) increased in the H-RES-HMB group. The relative abundance of *Escherichia-Shigella* (2.79%), *Rummeliibacillus* (0.12%), and that of *Terrisporobacter* (0.02%) decreased in the H-RES-HMB group (Fig. 4E).

### Metabolomics analysis of jejunum

**Fig 5:**
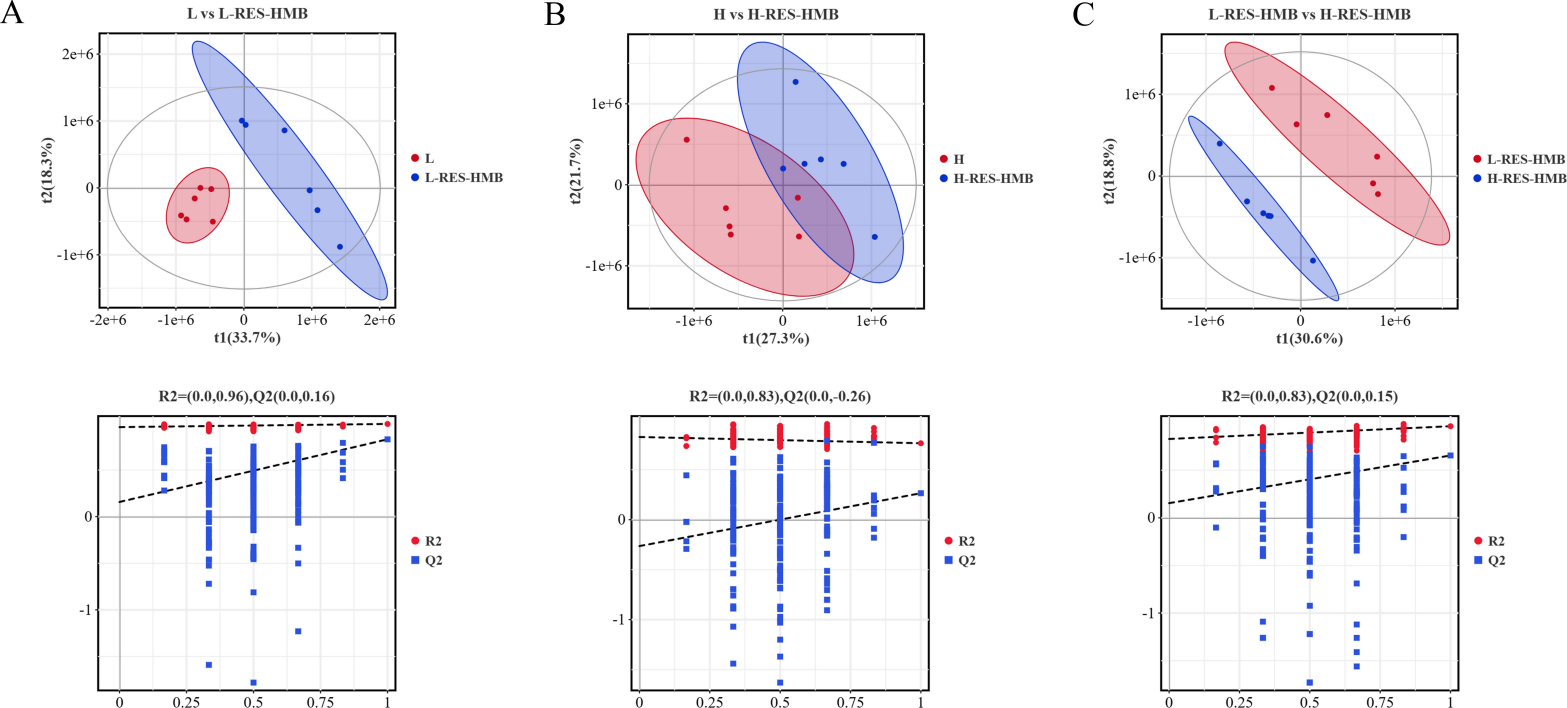
PLS-DA score plot and replacement test plot. (**A**) L vs L-RES-HMB group. (**B**) H vs H-RES-HMB group. (**C**) L-RES-HMB vs H-RES-HMB group.

Figure 5 presents the PLS-DA score plots and permutation test plots for the patterns of negative and positive ions between the four groups. The PLS-DA score plot exhibited good intra-group polymerization and inter-group separation between the L and L-RES-HMB groups (R2X = 0.582, R2Y = 0.994, and Q2 = 0.829; Fig. 5A), the H group and the H-RES-HMB group (R2X = 0.490, R2Y = 0.765, and Q2 = 0.265; Fig. 5B), as well as the L-RES-HMB and the H-RES-HMB groups (R2X = 0.494, R2Y = 0.962, and Q2 = 0.655; Fig. 5C).

**Fig 6:**
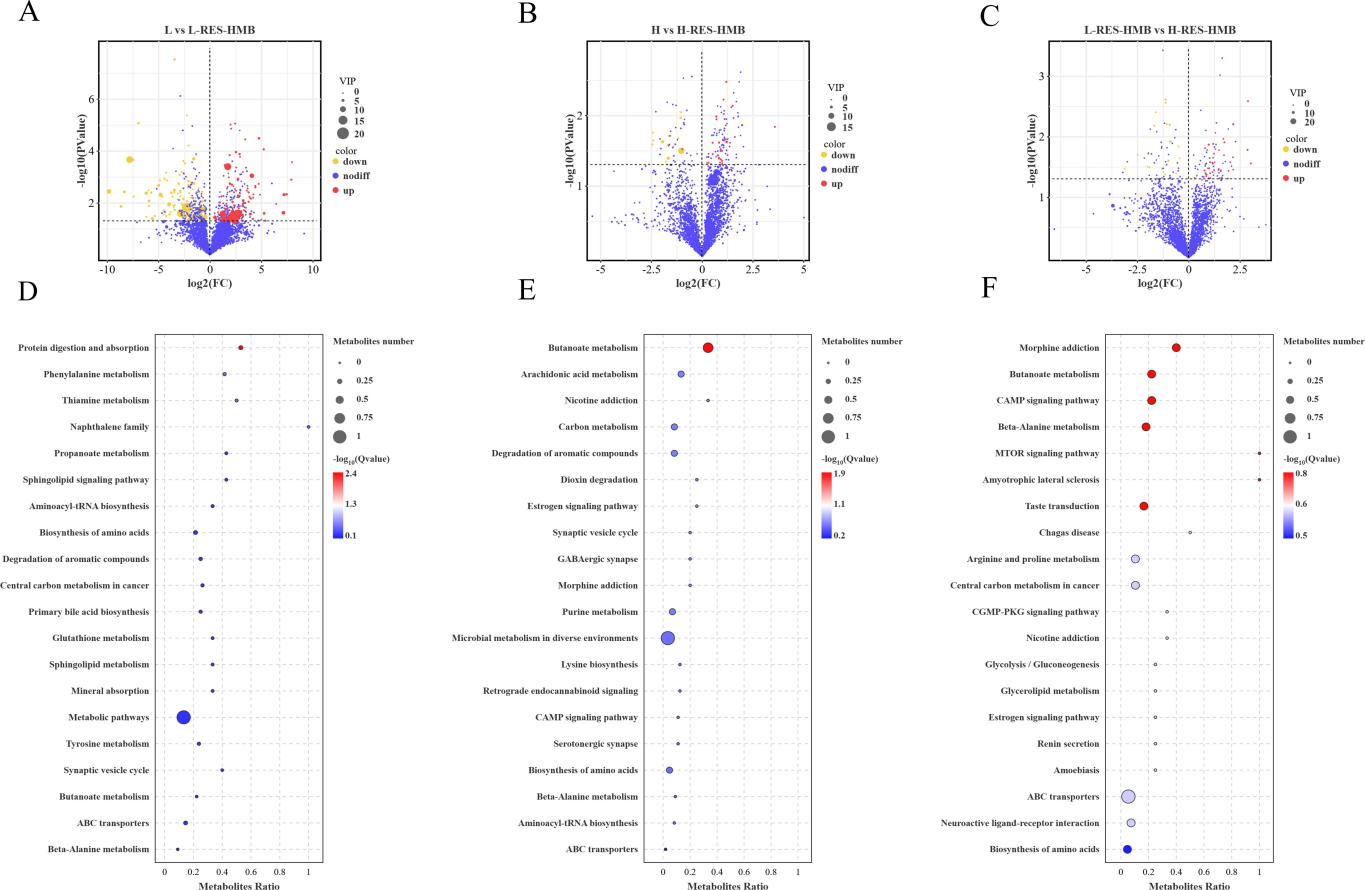
Volcano plots of (**A**) L vs L-RES-HMB group, (**B**) H vs H-RES-HMB group, (**C**) L-RES-HMB vs H-RES-HMB group DMs. (**D**) L vs L-RES-HMB group, (**E**) H vs H-RES-HMB group, and (**F**) L-RES-HMB vs H-RES-HMB group differential metabolite KEGG enrichment pathway. up: DMs significantly up-regulated; nodiff: no significant difference DMs; down: DMs significantly down-regulated.

After a thorough analysis of DMs, 307 DMs were identified (VIP > 1, *P* < 0.05). In the L and L-RES-HMB groups (Fig. 6A), 96 metabolites were upregulated, while 141 metabolites were downregulated. In the H and H-RES-HMB groups (Fig. 6B), 27 metabolites were upregulated, while 14 metabolites were downregulated. In the L-RES-HMB and H-RES-HMB groups, 32 metabolites were upregulated, while 23 metabolites were downregulated (Fig. 6C). Evaluation of the four major groups of DMs, eight metabolites were screened, including two organooxygen compounds (diacetyl and D-xylose), and three carboxylic acids and derivatives (L-arginine, isocitric acid, and His-Lys). The metabolite abundance of L-arginine and His-Lys was higher in the H-RES-HMB group, while that of diacetyl, D-xylose, and isocitric acid was lower in the H-RES-HMB group.

The enrichment analysis conducted on metabolic pathways indicated that the DMs identified in the L and L-RES-HMB cohorts were predominantly concentrated within pathways associated with protein digestion and absorption, amino acid biosynthesis, as well as general metabolic processes (refer to Fig. 6D). Conversely, the DMs observed in the H and H-RES-HMB groups were significantly enriched in pathways linked to butanoate metabolism, microbial metabolic activities across various environments, and amino acid biosynthesis (see Fig. 6E). Similarly, the DMs from both the L-RES-HMB and H-RES-HMB groups exhibited notable enrichment in pathways related to butanoate metabolism, beta-alanine metabolism, and the transport processes mediated by ATP-binding cassette (ABC) transporters (illustrated in Fig. 6F).

### Correlation analysis

#### Correlation study of SCFAs with digestive enzyme activity, antioxidant capacity, jejunum tissue morphology, immune response, and jejunum barrier function

**Fig 7:**
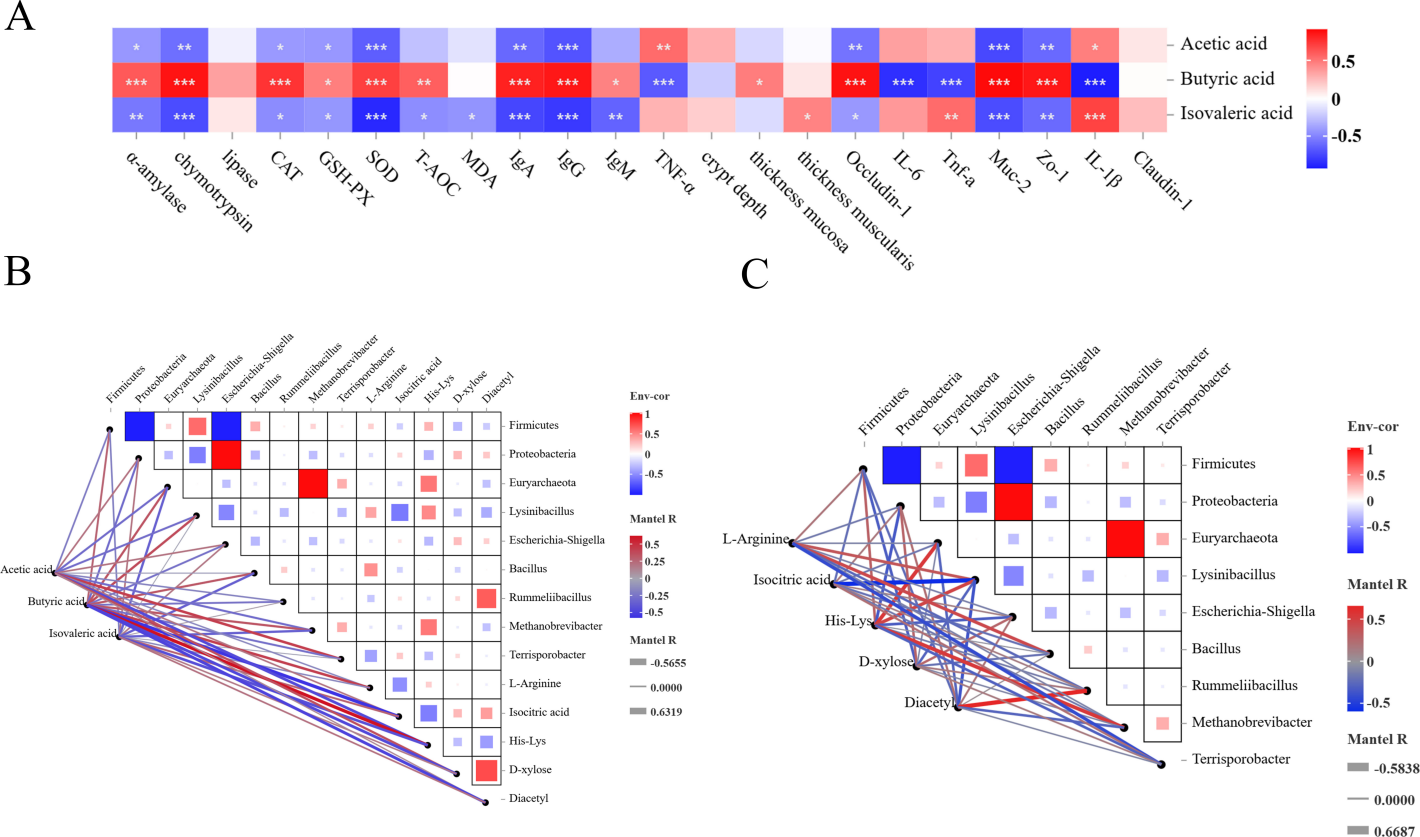
Generation correlation analysis. (**A**) Heat map of correlation between jejunal VFCAs (acetic acid, butyric acid, and isovalerate) and digestive enzyme activity, antioxidant indices, immune response, morphological parameters, and jejunal barrier. (**B**) Pearson correlation heat map of jejunum VFCAs (acetic acid, butyric acid, and isovaleric acid) with bacteria and metabolites. (**C**) Pearson correlation heat map of jejunal flora and metabolomics.

As shown in Fig. 7A, there existed a positive correlation between concentration of butyric acid and digestive enzyme activity (α-amylase and chymotrypsin), antioxidant capacity (CAT, GSH-PXH, SOD, and T-AOC), immune response (IgA and IgG), tissue morphology parameters (TM), and expression of genes related to jejunal barrier (OCLN, Muc-2, and ZO-1), and a negative correlation between immune response (TNF-α) and expression of jejunal barrier-related genes (IL-6, Tnf-α, and IL-1β). The concentrations of acetic acid and isovaleric acid showed a negative correlation with digestive enzyme activity (α-amylase and chymotrypsin), antioxidant capacity (SOD), immune response (IgA and IgG), and expression of genes related to jejunum barrier (Muc-2 and ZO-1).

#### Correlation study of SCFAs with jejunal microbiology and metabolomics

The concentration of butyric acid was positively correlated with *Firmicutes*, *Euryarchaeota*, *Lysinibacillus*, *Bacillus*, *Methanobrevibacter*, and His-Lys, and negatively correlated with isometric acid, D-xylose, and diacetyl (as shown in Fig. 7B).

#### Correlation study between microorganisms in the jejunum and metabolomics

L-Arginine was positively correlated with the abundance of *Lysinibacillus* and *Bacillus*, but a negative correlation with the abundance of *Terriporobacillus*. Isometric acid showed a negative correlation with the abundance of *Lysinibacillus* (as shown in Fig. 7C). His-Lys showed a positive correlation with the abundance of *Euryarchaeota*, *Lysinibacillus*, and *Methanobrevibacter*, but negative correlation with the abundance of *Proteobacteria* and *Terriporobacter*. Diacetyl showed a positive correlation with the abundance of *Rummeliibacillus*.

## DISCUSSION

Digestive enzymes are essential substances in the intestine that break down proteins, carbohydrates, lipids, and other nutrients into smaller, more digestible molecules. For instance, amylase, protease, and lipase are used for the digestion and absorption of intestinal nutrients (27). In this study, when Tibetan sheep were fed with a high-protein diet (14% CP), there was a significant increase in lipase level and no significant change in other indicators. Wang et al. found reduced digestive enzyme activity in pigs fed with a very low protein (12%) diet (28). Therefore, when the optimal supplemental dose of RES and HMB was added to the optimal diet (14% CP), the content of α-amylase and chymotrypsin increased significantly. The addition of RES and HMB increased the activities of α-amylase, chymotrypsin, and lipase of Tibetan sheep (17), consistent with the results of this study. Therefore, we believe that the addition of RES and HMB to a 14% protein diet can better enhance the digestive enzyme activity of jejunum contents. Currently, there are only a few studies on the combined addition of RES and HMB in diets, and the mechanism of action needs to be explored in the future.

Antioxidant activity reflects intestinal oxidative damage and metabolic activity of intestinal cells (29). CAT, GSH-PX, and SOD are the major antioxidant enzyme systems (30); excessive ROS is usually removed by antioxidant enzymes such as CAT, GSH-PX, and SOD. MDA is a commonly used indicator to assess the degree of peroxidation-induced tissue damage (31). When Tibetan sheep were fed a high protein diet (14% CP), the levels of CAT and T-AOC increased and MDA decreased. When RES and HMB were added to the optimal diet (14% CP), the activities of CAT, GSH-PX, and SOD were the highest, while that of MDA was lowest. The diet supplemented with RES exhibited significantly increased activities of CAT, GSH-PX, and SOD in intestinal tract, and the content of MDA decreased in jejunum mucosa of weaned piglets (32). Studies have also shown that RES can increase the activity of CAT, GSH-PX, and SOD in cells (33). Currently, there are only a few reports on the intestinal antioxidant effects of HMB, but studies have confirmed that CAT and GSH-PX activities in the jejunum of Tibetan sheep supplemented with RES and HMB are higher than those supplemented singly with RES or HMB (17), consistent with the results of this study. Therefore, we concluded that the addition of RES and HMB to a 14% protein diet can improve the activity of antioxidant enzymes in jejunum tissue better.

The immune system of animals is a key factor in the maintenance of relative stability of the internal environment. The immune markers, IgA, IgG, and IgM, play important roles in adaptive immunity, and IL-1β, IL-6, and TNF-α are the primary factors that promote cellular inflammation. RES exerts functions such as anti-inflammation and immunomodulatory activity, and HMB can improve immune function (34). This study showed that when Tibetan sheep were fed with a high protein diet (14% CP), the level of IgG increased, while IL-6, IL-1β, and TNF-α levels decreased. When RES and HMB were supplemented to the optimal diet (14% CP), the IgA, IgG, and IgM activities in the jejunum of the H-RES-HMB group were higher compared to those of the other three groups. Not only can polyphenolic plants increase the production of IgA (35), but leucine supplementation can also increase the expression concentrations of IgA, IgM, and IL-17, consistent with the results of this experiment (36). In this study, the H-RES-HMB group exhibited a decrease in the TNF-α content of jejunum. Several studies have demonstrated that this phenolic compound (RES) can be used to treat and prevent tumors, block uncontrolled cell growth, and exert anti-inflammatory properties by reducing the activities of pro-inflammatory cytokines (IL-1β, IL-6, and TNF-α) (37). Gao et al. found that the administration of RES reduced TNF-α levels (38), consistent with the results of this experiment. Therefore, adding RES and HMB to the 14% protein diet can further enhance the immune activity in jejunum tissue.

The effects of RES and HMB on the intestinal tract have received increasing attention. The development of the intestine, an organ with a major role in the digestion and absorption of dietary components, is influenced by dietary intervention. VH and CD were used to assess intestinal integrity (39), and the ratio of VH to CD was used to assess intestinal influence on disease or dietary response (40). Jejunal histological parameters showed that feeding a high protein diet (14% CP) increased VH, VW, CD, TM, and MT, and a low protein level reduced rumen mucosal thickness (41). In this study, the addition of RES and HMB to a 14% protein diet significantly decreased the CD of jejunum, and the ratio of TM, MT, and VH to CD tended to increase. In this regard, several studies have shown that supplementation with RES or HMB could increase the VH of jejunum and the ratio of VH to CD and reduce the CD (13, 32, 42). The combination of RES can reduce the damage to the gastrointestinal mucosa, crypt, and muscle layer (43), and HMB enhances the mucosal thickness of the ileum (44). Studies have shown that the combined addition of RES and HMB significantly increases the thickness of mucosa compared to the control group (17), consistent with the results of this study. Therefore, supplementation of RES and HMB to a 14% protein diet has more positive effects on jejunum tissue morphology. At present, there are many studies on the addition of RES and HMB separately, but their synergistic effects need to be further proved.

The integrity of the intestinal barrier (intestinal mucosa, tight junctions, and intestinal epithelial cells [IEC]) is a safety barrier against harmful substances entering the body. *ZO-1* and *OCLN* are two tight junction proteins in intestinal mucosa that play a key role in the intestinal epithelial barrier formation and maintenance (45). *ZO-1* is the first discovered tight junction protein, and its change might lead to the disturbance of the small intestinal mucosal tight junction barrier of the small intestine, which results in enhanced intestinal permeability (46), and *OCLN* is an important regulator of immune-mediated inflammatory bowel disease (47). Muc-2 is the first described gene of the intestinal mucin secreted in an organ system (48). Pro-inflammatory cytokines (such as IL-6 and IL-1β) are important indicators that participate in immune and inflammatory responses (49). One study of dietary protein levels on intestinal function and inflammation in pigs reported elevated TER and healthier intestinal barrier was when fed a high-protein diet (26% CP) (50). The optimal dietary protein level can enhance the function of intestinal immune barrier and up-regulate the tight junction complex (51). In this study, feeding the optimal protein level (14% CP) diet enhanced the expression of *OCLN* and *ZO-1* of jejunum barrier function and decreased IL-6 expression. Studies have confirmed that RES treatment not only enhances the mRNA expression of occludin and *ZO-1* in the intestinal barrier function (33, 52), but also attenuates inflammatory diseases of the intestine by decreasing the expression of pro-inflammatory factors, such as IL-6 and IL-1β (53). A study on zearalenone (ZEA)-induced intestinal mucosal injury in mice reported that RES significantly increased the expression of the *Muc-2* gene (54). Studies have also found that HMB inhibits apoptosis by suppressing IL-6, IL-1β, and other genes in the nuclear factor κB (NF-κB) pathway (55). Other studies have further shown that dietary leucine can enhance the expression of intestinal barrier-related functions *ZO-1* and *occludin* (56). These findings are consistent with our experimental results. In the current study, feeding the 14% protein diet supplemented with RES and HMB enhanced the mRNA expression of *OCLN, Muc-2,* and *ZO-1* in the jejunum of Tibetan sheep, decreased the expression of IL-6 and IL-1β, improved the jejunal barrier function, and promoted the integrity of the intestinal barrier.

SCFAs are an important fuel for IEC and have a role in enhancing intestinal barrier function. In particular, butyrate is reflected in intestinal and immunomodulatory functions. In this study, the dietary addition of RES and HMB significantly increased the concentrations of acetic acid, butyric acid, and isovaleric acid. Previous studies revealed that RES altered the concentrations of N-butyric acid and isobutyric acid in the intestinal SCFAs in the gut of mice (57), possibly due to the increased abundance of bacteria producing SCFAs induced by RES (58). Zheng et al. found that supplementation with HMB increased the concentration of all SCFAs except propionate (13). The results of this study are consistent with those reported earlier. In this study, the 14% protein diet significantly increased butyric acid concentration. A study on pigs fed with diets having different protein levels confirmed that pigs on a 14% protein diet had higher concentrations of total SCFAs than other groups (59). This finding is consistent with our results, which showed that the addition of RES and HMB to a 14% protein diet significantly enhanced the concentration of butyric acid, which, along with its derivatives, not only improves intestinal function but also plays an important role in the immune system, intestinal inflammation, and oxidative stress. Studies have further shown two important mechanisms of butyrate for inhibiting the activation of NF-κB and histone deacetylation (60), consequently generally enhancing intestinal immune function through G protein-mediated signaling pathways and reducing the expression of inflammatory factors by inhibiting histone deacetylase (61); thereby, the expression of IL-6, IL-1β, and TNF-α decreased. In this study, we observed a positive correlation of butyric acid concentration with α-amylase, chymotrypsin, CAT, GSH-PXH, SOD, T-AOC, IgA, IgG, TM, *OCLN, Muc-2*, and *Zo-1*, but negative correlation with IL-6, Tnf-α, and IL-1β. Therefore, the addition of RES and HMB to a 14% protein diet increased butyric acid concentration, thereby enhancing jejunum antioxidant capacity and changes in the immune system and intestinal function.

Intestinal flora plays a crucial role in nutrient absorption, antioxidant capacity, and immune function, while also maintaining the integrity of the gastrointestinal barrier. It establishes a close relationship with the host, supporting the overall gut health (62). In this study, the addition of RES and HMB to the optimal protein (14%) diet increased the relative abundance of *Firmicutes*, *Bacillus,* and *Methanobrevibacter* in the jejunum. *Firmicutes* interact with intestinal mucosa to promote balanced metabolism of the body (63). *Bacillus* secretes a variety of antibacterial substances, having high stability and broad-spectrum antimicrobial activity (64, 65). Some studies have reported that *B. pumilus* supplementation decreased the expression of IL-6, IL-1β, and TNF-α and increased jejunum height in mice (66, 67), while others have reported the effects of different strains of *Bacillus* on immune response and intestinal integrity (68–70), while some studies have confirmed that different *Bacilli* alter the composition of the intestinal microbiota and SCFA content, thereby improving diseases caused by intestinal inflammation (71, 72). Our research further indicates that the abundance of *Firmicutes* and *Bacillus* is positively correlated with butyric acid concentration. The gene encoding NOX was identified from *Methanobrevibacter smithii* (NOX ms), while NADH oxidase (NOX) is a key intermediate that protects animals from oxidative stress and maintains the NAD/NADH balance (73). In this study, the addition of RES and HMB to a 14% protein diet reduced the relative abundance of *Proteobacteria* and *Escherichia*-Shigella. *Proteobacteria* is mainly associated with metabolic disorders and inflammatory bowel disease (74), and lipopolysaccharides are present in its outer membrane. *Proteobacteria* promote inflammation of the intestinal mucosal wall, leading to the disruption of the intestinal epithelial cell barrier (75). *Escherichia-Shigella* is also one of the main microorganisms; studies have revealed that the abundance of *Proteobacteria* and *Escherichia-Shigella* is increased, thereby increasing IL-6 and IL-1β expression (76). The results of this study indicate that RES and HMB inhibited the abundance of *Proteobacteria and Escherichia Shigella*. In our study, the abundance of *Proteobacteria* and *Escherichia-Shigella* was negatively correlated with butyric acid. Therefore, we suggest that the addition of RES and HMB to the 14% protein diet can improve the activity of intestinal digestive enzymes, oxidative stress, immune response, intestinal barrier, and SCFA by regulating intestinal microbiota composition.

Analysis of the differential pathways of the metabolome and important metabolites confirmed that significant changes in the jejunal content metabolism were greatly influenced by dietary protein levels and RES and HMB additives. These DMs are mainly enriched in the metabolic pathways of biosynthesis of amino acids, ABC transporters, and butanoate metabolism. Amino acids, as substrates for protein biosynthesis, are important regulators of major metabolic pathways (77). In this study, the addition of RES and HMB to the 14% protein diet enhanced the concentration of L-arginine, which is associated with the biosynthesis of amino acids, and decreased the concentration of isocitric acid. L-Arginine acts as a substrate for intestinal and microbial cells (78); studies have confirmed that supplementation of L-arginine in the diet not only enhanced GSH-PX activity and T-AOC of the chicken intestine but also decreased IL-1β expression and increased the abundance of Firmicutes, which improved intestinal antioxidant capacity, immune response, and intestinal barrier function (79, 80). Furthermore, Jabecka et al. found that an increase in L-arginine concentration stimulated NO biosynthesis, thereby reducing oxidative stress (81). Isometric acid is an intermediate in the citric acid cycle, which has antioxidant and anti-stress properties (82); however, too much isocitric acid will stimulate gastrointestinal metabolic disorders, resulting in acidosis. Therefore, moderate amounts of isocitric acid help metabolic balance. Similarly, our research indicates that L-arginine is positively correlated with the abundance of *Firmicutes* and *Bacillus*. ABC transporters are integral membrane components that make up the largest integrated membrane proteins and are involved in functions such as nutrient absorption and immunity (83). Research has found that changes in the activity of ABC transporters can affect the likelihood of developing intestinal inflammation and colon cancer, thereby improving gut health (84). In this study, the addition of RES and HMB to the 14% protein diet increased the concentration of His-Lys associated with ABC transporters and decreased the concentration of D-xylose. His and Lys are the two amino acids with the most significant antioxidant properties that can increase the activity of GSH S-transferase (85), a multifunctional enzyme that protects cells from oxidative stress in various diseases (86). His-Lys also affected IgA and VH of porcine jejunum and improved intestinal health (87). Therefore, His-Lys protects tissue morphological development and enhances immune response. D-xylose, a five-carbon sugar, is one of the most abundant natural carbohydrates and serves as an energy source for cells. Research reveals that D-xylose is the key intermediate in ethanol production (88). Therefore, lower D-xylose levels may reduce bacterial fermentation products and lower intestinal inflammation. One study on mice demonstrated that improved colon inflammation was followed by a significant decline in serum D-xylose levels (89). In our study, we observed a positive correlation of His-Lys with the abundance of *Firmicutes* and *Methanobrevibacter*. Butyrate is an important metabolic substrate for butanoate metabolism and plays a crucial role in the gut (90). Butyrate significantly increases the expression levels of tight junction protein mRNA in the jejunum. It reduces intestinal inflammation and maintains cellular homeostasis. Butyrate also improves alterations in intestinal barrier function (91). A study of patients with ulcerative colitis revealed that abnormal butyrate metabolism directly leads to impaired intestinal barrier function and affects intestinal inflammation (92). This highlights the key role of butyrate metabolism in maintaining intestinal homeostasis. Gamma-aminobutyric acid (GABA) is a non-protein amino acid that regulates various physiological functions (93). The metabolic precursor and product of GABA)is gamma hydroxybutyric acid (GHB), and the metabolism of GHB produces NADPH, which is an important antioxidant cofactor (94). Not only that, GHB metabolism also produces succinate, a cyclic intermediate of the tricarboxylic acid cycle, which provides energy for cells and synthesizes glutamate (95). This study promoted the increase of butyrate concentration by regulating GABA and succinate metabolites in butanoate metabolism. Additionally, the addition of RES and HMB to a 14% protein diet decreased the concentration of diacetyl associated with butanoate metabolism. Diacetyl is a key metabolite marker for various cancers (96), which can impair respiratory damage in humans or test animals, and also disrupt the health of the intestinal tract (97, 98), whereas butyric acid can inhibit the diacetyl production by decreasing the activity of diacetyl reductase (99). In this study, diacetyl showed a negative correlation with *Firmicutes*, *Methanobrevibacter*, and butyric acid. Therefore, the changes in metabolites identified in this study may positively impact the intestinal health of Tibetan sheep.

The Pearson correlation analysis between microbiota and metabolome showed positive correlation of L-arginine with the abundance of *Bacillus*; His-Lys was positively correlated with the abundance of *Methanobrevibacter* and negatively correlated with the abundance of *Proteobacteria*. Diacetyl was negatively correlated with the abundance of *Firmicutes* and *Methanobrevibacter*. We speculate that the biosynthesis of amino acids, ABC transporters, and butanoate metabolism in the L group, L-RES-HMB group, H group, and H-RES-HMB group is related to changes in the abundance of *Bacillus*, *Firmicutes*, *Methanobrevibacter*, and *Proteobacteria*.

### Conclusion

**Fig 8:**
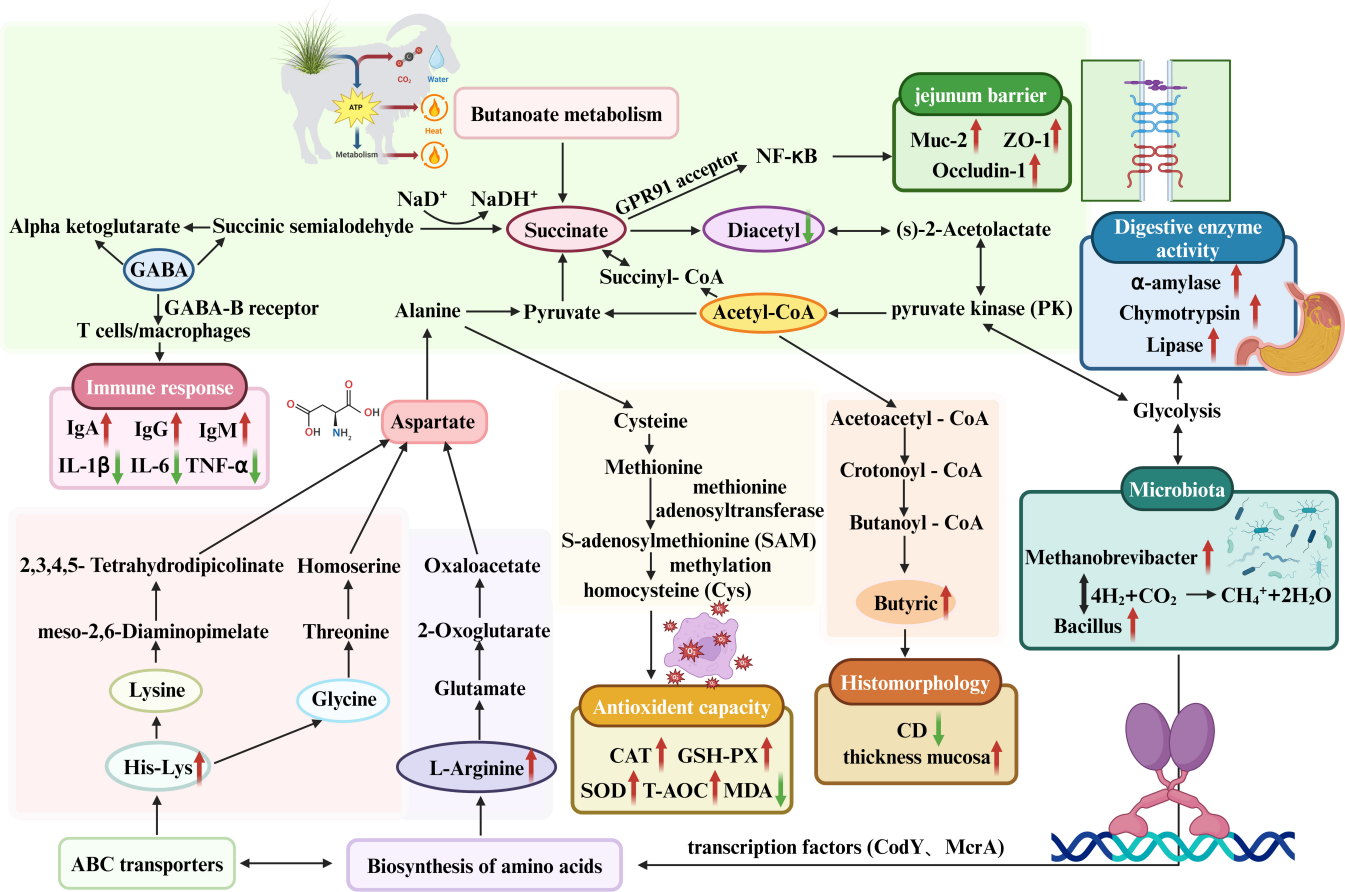
The schematic diagram shows that adding RES and HMB to diets with different protein levels can increase butyrate concentration by regulating microbial composition and metabolites, thereby altering intestinal digestive enzyme activity, antioxidant capacity, immune response, intestinal development integrity, and barrier function.

In conclusion, the results of this study indicated that the 12.9% protein diet improved the morphological integrity and barrier function of the jejunum in Tibetan sheep compared to the 11.5% protein diet, with the concomitant significant increase in butyric acid level. In addition, the supplementation of RES and HMB to the 14% protein diet could regulate microorganisms (*Firmicutes, Methanobrevibacter,* and *Bacillus*), biosynthesis of amino acids (L-Arginine), ABC transporters (His-Lys), and butanoate metabolism (GABA and Succinate), thereby promoting the increase of butyrate concentration and subsequently modulating gut digestive enzyme activity, antioxidant capacity, immune response, intestinal development integrity, and barrier function. The mechanism contained in the article is shown in Fig. 8. This study demonstrates that the interaction among RES, HMB, and protein enhances intestinal function and gut health in Tibetan sheep. These results contribute valuable insights to the field of intestinal health research and offer an innovative theoretical framework to support healthy breeding and production.

## Data Availability

The data sets presented in this study can be found in NCBI SRA (accession no. PRJNA1127188) and OMIX, China National Center for Bioinformation/Beijing Institute of Genomics, Chinese Academy of Sciences (https://ngdc.cncb.ac.cn/omix) under accession no. OMIX014071.

## SUPPLEMENTAL MATERIAL

Table S1 spectrum.02843-25-s0001.docx Jejunal primer sequences.

ASM does not own the copyrights to Supplemental Material that may be linked to, or accessed through, an article. The authors have granted ASM a non-exclusive, world-wide license to publish the Supplemental Material files. Please contact the corresponding author directly for reuse.

## References

[1] Li F, Hitch TCA, Chen Y, Creevey CJ, Guan LL. 2019. Comparative metagenomic and metatranscriptomic analyses reveal the breed effect on the rumen microbiome and its associations with feed efficiency in beef cattle. Microbiome 7:6. doi:10.1186/s40168-019-0618-5 30642389 PMC6332916

[2] Wang X, Zhang Q, Guo T, Li S, Jia Y, Xu S. 2025. Multi-omics analysis reveals host-microbe interactions driving divergent energy allocation strategies in Tibetan sheep under cold-season feeding regimes. J Anim Sci Biotechnol 16:122. doi:10.1186/s40104-025-01259-w 40890867 PMC12403966

[3] Wei X, Dong Z, Cheng F, Shi H, Zhou X, Li B, Wang L, Wang W, Zhang J. 2021. Seasonal diets supersede host species in shaping the distal gut microbiota of Yaks and Tibetan sheep. Sci Rep 11. doi:10.1038/s41598-021-99351-4 PMC8604981 34799677

[4] Liu J, Han L, Hou S, Gui L, Yuan Z, Sun S, Wang Z, Yang B. 2024. Integrated metabolome and microbiome analysis reveals the effect of rumen-protected sulfur-containing amino acids on the meat quality of Tibetan sheep meat. Front Microbiol 15. doi:10.3389/fmicb.2024.1345388 PMC10883651 38389537

[5] Zhang F, Wu Z, Zhang Y, Su Q, Zhu K, Chen X, Hou S, Gui L. 2025. Different lysine-to-methionine ratios in a low-protein diet affect the microbiome and metabolome, influencing the jejunal barrier function in Tibetan sheep. Front Microbiol 16:1441143. doi:10.3389/fmicb.2025.1441143 40012772 PMC11861081

[6] Florence AT. 2005. Nanoparticle uptake by the oral route: fulfilling its potential? Drug Discov Today Technol 2:75–81. doi:10.1016/j.ddtec.2005.05.019 24981758

[7] Oz HS, Chen TS, McClain CJ, de Villiers WJS. 2005. Antioxidants as novel therapy in a murine model of colitis. J Nutr Biochem 16:297–304. doi:10.1016/j.jnutbio.2004.09.007 15866230

[8] Chiu HF, Venkatakrishnan K, Golovinskaia O, Wang CK. 2021. Gastroprotective effects of polyphenols against various gastro-intestinal disorders: a mini-review with special focus on clinical evidence. Molecules 26:2090. doi:10.3390/molecules26072090 33917379 PMC8038706

[9] Sessa M, Balestrieri ML, Ferrari G, Servillo L, Castaldo D, D’Onofrio N, Donsì F, Tsao R. 2014. Bioavailability of encapsulated resveratrol into nanoemulsion-based delivery systems. Food Chem 147:42–50. doi:10.1016/j.foodchem.2013.09.088 24206683

[10] Zhang C, Zhao XH, Yang L, Chen XY, Jiang RS, Jin SH, Geng ZY. 2017. Resveratrol alleviates heat stress-induced impairment of intestinal morphology, microflora, and barrier integrity in broilers. Poult Sci 96:4325–4332. doi:10.3382/ps/pex266 29053872

[11] Meng Q, Li J, Wang C, Shan A. 2023. Biological function of resveratrol and its application in animal production: a review. J Animal Sci Biotechnol 14:25. doi:10.1186/s40104-022-00822-z PMC9921422 36765425

[12] Kim J, Kim EK. 2020. Nutritional strategies to optimize performanceand recovery in rowing athletes. Nutrients 12:1685. doi:10.3390/nu12061685 32516908 PMC7352678

[13] Zheng C, Song B, Duan Y, Zhong Y, Yan Z, Zhang S, Li F. 2020. Dietary β-hydroxy-β-methylbutyrate improves intestinal function in weaned piglets after lipopolysaccharide challenge. Nutrition 78:110839. doi:10.1016/j.nut.2020.110839 32540677

[14] Wójcik R, Ząbek K, Małaczewska J, Milewski S, Kaczorek-Łukowska E. 2019. The effects of β-hydroxy-β-methylbutyrate (HMB) on chemotaxis, phagocytosis, and oxidative burst of peripheral blood granulocytes and monocytes in goats. Animals (Basel) 9:1031. doi:10.3390/ani9121031 31779122 PMC6940930

[15] Gan J, Ji Q, Su Q, Hou S, Gui L. 2024. Resveratrol and β-hydroxy-β-methylbutyric acid supplementation promotes ileal development and digestive function by altering microbial community abundance and metabolites in Tibetan sheep. Front Vet Sci 11:1470992. doi:10.3389/fvets.2024.1470992 39723186 PMC11668758

[16] Zhang Y, Ji Q, Gui L, Gebeyew K, Hou S, Wang Z, Han L, Yang C. 2025. The influence of resveratrol and β-Hydroxy-β-methyl butyric acid supplementation alone or in combination on the development and health of the duodenum in Tibetan sheep. Front Microbiol 16:1612102. doi:10.3389/fmicb.2025.1612102 40698187 PMC12279740

[17] Ji Q, Zhang F, Zhang Y, Su Q, He T, Hou S, Gui L. 2024. Multi-omics revealed resveratrol and β-hydroxy-β-methyl butyric acid alone or in combination improved the jejunal function in tibetan sheep. Antioxidants (Basel) 13:892. doi:10.3390/antiox13080892 39199138 PMC11351831

[18] Chen S, Zhou Y, Chen Y, Gu J. 2018. Fastp: an ultra-fast all-in-one FASTQ preprocessor. Bioinformatics 34:i884–i890. doi:10.1093/bioinformatics/bty560 30423086 PMC6129281

[19] Magoč T, Salzberg SL. 2011. FLASH: fast length adjustment of short reads to improve genome assemblies. Bioinformatics 27:2957–2963. doi:10.1093/bioinformatics/btr507 21903629 PMC3198573

[20] Edgar RC. 2013. UPARSE: highly accurate OTU sequences from microbial amplicon reads. Nat Methods 10:996–998. doi:10.1038/nmeth.2604 23955772

[21] Edgar RC, Haas BJ, Clemente JC, Quince C, Knight R. 2011. UCHIME improves sensitivity and speed of chimera detection. Bioinformatics 27:2194–2200. doi:10.1093/bioinformatics/btr381 21700674 PMC3150044

[22] Wang Q, Garrity GM, Tiedje JM, Cole JR. 2007. Naive Bayesian classifier for rapid assignment of rRNA sequences into the new bacterial taxonomy. Appl Environ Microbiol 73:5261–5267. doi:10.1128/AEM.00062-07 17586664 PMC1950982

[23] Pruesse E, Quast C, Knittel K, Fuchs BM, Ludwig W, Peplies J, Glöckner FO. 2007. SILVA: a comprehensive online resource for quality checked and aligned ribosomal RNA sequence data compatible with ARB. Nucleic Acids Res 35:7188–7196. doi:10.1093/nar/gkm864 17947321 PMC2175337

[24] Warnes GR. 2007. gmodels: various R programming tools for model fitting

[25] Thevenot EA. 2016. PCA, PLS(-DA) and OPLS(-DA) for multivariate analysis and feature selection of omics data

[26] Revelle W. 2010. Package “psych”

[27] Rideau N, Nitzan Z, Mongin P. 1983. Activities of amylase, trypsin and lipase in the pancreas and small intestine of the laying hen during egg formation. Br Poult Sci 24:1–9. doi:10.1080/00071668308416707 6187420

[28] Wang Y, Han S, Zhou J, Li P, Wang G, Yu H, Cai S, Zeng X, Johnston LJ, Levesque CL, Qiao S. 2020. Effects of dietary crude protein level and N-carbamylglutamate supplementation on nutrient digestibility and digestive enzyme activity of jejunum in growing pigs. J Anim Sci 98:skaa088. doi:10.1093/jas/skaa088 32201878 PMC7149547

[29] Chen Z, Wang G, Wang W, Wang X, Huang Y, Jia J, Gao Q, Xu H, He L, Xu Y, Liu Z, Sun J, Li C. 2024. Relationship between jejunum ATPase activity and antioxidant function on the growth performance, feed conversion efficiency, and jejunum microbiota in Hu sheep (Ovis aries). BMC Vet Res 20:242. doi:10.1186/s12917-024-04100-0 38831422 PMC11149274

[30] Bin-Jumah MN, Al-Huqail AA, Abdelnaeim N, Kamel M, Fouda MMA, Abulmeaty MMA, Saadeldin IM, Abdel-Daim MM. 2021. Potential protective effects of Spirulina platensis on liver, kidney, and brain acrylamide toxicity in rats. Environ Sci Pollut Res Int 28:26653–26663. doi:10.1007/s11356-021-12422-x 33492591

[31] Fei M, Xie Q, Zou Y, He R, Zhang Y, Wang J, Bo L, Li J, Deng X. 2016. Alpha-lipoic acid protects mice against concanavalin A-induced hepatitis by modulating cytokine secretion and reducing reactive oxygen species generation. Int Immunopharmacol 35:53–60. doi:10.1016/j.intimp.2016.03.023 27018751

[32] Xun W, Fu Q, Shi L, Cao T, Jiang H, Ma Z. 2021. Resveratrol protects intestinal integrity, alleviates intestinal inflammation and oxidative stress by modulating AhR/Nrf2 pathways in weaned piglets challenged with diquat. Int Immunopharmacol 99:107989. doi:10.1016/j.intimp.2021.107989 34303281

[33] Zhuang Y, Wu H, Wang X, He J, He S, Yin Y. 2019. Resveratrol attenuates oxidative stress-induced intestinal barrier injury through PI3K/Akt-mediated Nrf2 signaling pathway. Oxid Med Cell Longev 2019:7591840. doi:10.1155/2019/7591840 31885814 PMC6915002

[34] Zabek K, Wojcik R, Milewski S, Malaczewska J, Tanski Z, Siwicki AK. 2016. Effect of β-hydroxy-β-methylbutyrate acid on meat performance traits and selected indicators of humoral immunity in goats. Jpn J Vet Res 64:247–256. doi:10.14943/jjvr.64.4.247 29786174

[35] Fujiki T, Shinozaki R, Udono M, Katakura Y. 2022. Identification and functional evaluation of polyphenols that induce regulatory T cells. Nutrients 14:2862. doi:10.3390/nu14142862 35889819 PMC9318754

[36] Gatnau R, Zimmerman DR, Nissen SL, Wannemuehler M, Ewan RC. 1995. Effects of excess dietary leucine and leucine catabolites on growth and immune responses in weanling pigs. J Anim Sci 73:159–165. doi:10.2527/1995.731159x 7601729

[37] Nutakul W, Sobers HS, Qiu P, Dong P, Decker EA, McClements DJ, Xiao H. 2011. Inhibitory effects of resveratrol and pterostilbene on human colon cancer cells: a side-by-side comparison. J Agric Food Chem 59:10964–10970. doi:10.1021/jf202846b 21936500 PMC3201709

[38] Gao X, Deeb D, Media J, Divine G, Jiang H, Chapman RA, Gautam SC. 2003. Immunomodulatory activity of resveratrol: discrepant in vitro and in vivo immunological effects. Biochem Pharmacol 66:2427–2435. doi:10.1016/j.bcp.2003.08.008 14637200

[39] Tomaszewska E, Świątkiewicz S, Arczewska-Włosek A, Wojtysiak D, Dobrowolski P, Domaradzki P, Puzio I, Rudyk H, Brezvyn O, Muszyński S. 2024. ß-Hydroxy-ß-methylbutyrate: a feed supplement influencing performance, bone metabolism, intestinal morphology, and muscle quality of laying hens: a preliminary one-point study. Poult Sci 103:103597. doi:10.1016/j.psj.2024.103597 38471225 PMC11067770

[40] Kwon O, Han TS, Son MY. 2020. Intestinal morphogenesis in development, regeneration, and disease: the potential utility of intestinal organoids for studying compartmentalization of the crypt-villus structure. Front Cell Dev Biol 8:593969. doi:10.3389/fcell.2020.593969 33195268 PMC7644937

[41] Cui K, Qi M, Wang S, Diao Q, Zhang N. 2019. Dietary energy and protein levels influenced the growth performance, ruminal morphology and fermentation and microbial diversity of lambs. Sci Rep 9:16612. doi:10.1038/s41598-019-53279-y 31719633 PMC6851105

[42] Suad KA, Al-Shamire JSH, Dhyaa AA. 2018. Histological and biochemical evaluation of supplementing broiler diet with β-hydroxy-methyl butyrate calcium (β-HMB-Ca). Iran J Vet Res 19:27–34. doi:10.22099/ijvr.2018.4764 29805459 PMC5960769

[43] Donald EL, Stojanovska L, Apostolopoulos V, Nurgali K. 2017. Resveratrol alleviates oxidative damage in enteric neurons and associated gastrointestinal dysfunction caused by chemotherapeutic agent oxaliplatin. Maturitas 105:100–106. doi:10.1016/j.maturitas.2017.05.010 28545905

[44] Kop Bozbay C, Yılmaz B, Ocak N. 2024. Beta-hydroxy-β-methyl butyrate-supplemented diet for broiler chickens is more conducive to dietary protein reduction than a leucine-supplemented diet until 21 days old. J Sci Food Agric 104:1450–1457. doi:10.1002/jsfa.13023 37800278

[45] Kuo W, Odenwald MA, Turner JR, Zuo L. 2022. Tight junction proteins occludin and ZO‐1 as regulators of epithelial proliferation and survival. Ann N Y Acad Sci 1514:21–33. doi:10.1111/nyas.14798 35580994 PMC9427709

[46] Hamada K, Shitara Y, Sekine S, Horie T. 2010. Zonula occludens-1 alterations and enhanced intestinal permeability in methotrexate-treated rats. Cancer Chemother Pharmacol 66:1031–1038. doi:10.1007/s00280-010-1253-9 20119715

[47] Raju P, Shashikanth N, Tsai P-Y, Pongkorpsakol P, Chanez-Paredes S, Steinhagen PR, Kuo W-T, Singh G, Tsukita S, Turner JR. 2020. Inactivation of paracellular cation-selective claudin-2 channels attenuates immune-mediated experimental colitis in mice. J Clin Invest 130:5197–5208. doi:10.1172/JCI138697 32516134 PMC7524482

[48] Toribara NW, Gum JR Jr, Culhane PJ, Lagace RE, Hicks JW, Petersen GM, Kim YS. 1991. MUC-2 human small intestinal mucin gene structure. Repeated arrays and polymorphism. J Clin Invest 88:1005–1013. doi:10.1172/JCI115360 1885763 PMC295506

[49] Li J, Chen C, Gao L, Wang L, Wang W, Zhang J, Gong Z, Wang J, Guo Y. 2023. Analysis of histopathology and changes of major cytokines in the lesions caused by Mycoplasma ovipneumoniae infection. BMC Vet Res 19:273. doi:10.1186/s12917-023-03829-4 38102682 PMC10722778

[50] Pearce SC, Nisley MJ, Kerr BJ, Sparks C, Gabler NK. 2024. Effects of dietary protein level on intestinal function and inflammation in nursery pigs. J Anim Sci 102:skae077. doi:10.1093/jas/skae077 38504643 PMC11015048

[51] Xu J, Wu P, Jiang W-D, Liu Y, Jiang J, Kuang S-Y, Tang L, Tang W-N, Zhang Y-A, Zhou X-Q, Feng L. 2016. Optimal dietary protein level improved growth, disease resistance, intestinal immune and physical barrier function of young grass carp (Ctenopharyngodon idella). Fish Shellfish Immunol 55:64–87. doi:10.1016/j.fsi.2016.05.021 27211261

[52] Song X, Liu L, Peng S, Liu T, Chen Y, Jia R, Zou Y, Li L, Zhao X, Liang X, Tang H, Yin Z. 2022. Resveratrol regulates intestinal barrier function in cyclophosphamide-induced immunosuppressed mice. J Sci Food Agric 102:1205–1215. doi:10.1002/jsfa.11458 34346509

[53] Yu X, Wang Y, Xu Y, Li X, Zhang J, Su Y, Guo L. 2024. Resveratrol attenuates intestinal epithelial barrier dysfunction via Nrf2/HO-1 pathway in dextran sulfate sodium-induced Caco-2 cells. Immun Inflamm Dis 12:e1193. doi:10.1002/iid3.1193 38372468 PMC10875904

[54] Xia S, Yan C, Gu J, Yuan Y, Zou H, Liu Z, Bian J. 2024. Resveratrol alleviates zearalenone-induced intestinal dysfunction in mice through the NF-κB/Nrf2/HO-1 signalling pathway. Foods 13:1217. doi:10.3390/foods13081217 38672890 PMC11049466

[55] Zheng J, Li B, Yan Y, Huang X, Zhang E. 2022. β-hydroxy-β-methylbutyric acid promotes repair of sheep myoblast injury by inhibiting IL-17/NF-κB signaling. Int J Mol Sci 24:444. doi:10.3390/ijms24010444 36613892 PMC9820147

[56] Zhao J, Zhao Y, Liu H, Cao Q, Feng L, Zhang Z, Jiang W, Wu P, Liu Y, Luo W, Huang X, Jiang J. 2023. Dietary leucine improves fish intestinal barrier function by increasing humoral immunity, antioxidant capacity, and tight junction. Int J Mol Sci 24:4716. doi:10.3390/ijms24054716 36902147 PMC10003359

[57] Alrafas HR, Busbee PB, Chitrala KN, Nagarkatti M, Nagarkatti P. 2020. Alterations in the gut microbiome and suppression of histone deacetylases by resveratrol are associated with attenuation of colonic inflammation and protection against colorectal cancer. J Clin Med 9:1796. doi:10.3390/jcm9061796 32526927 PMC7355848

[58] Wang P, Wang J, Li D, Ke W, Chen F, Hu X. 2020. Targeting the gut microbiota with resveratrol: a demonstration of novel evidence for the management of hepatic steatosis. J Nutr Biochem 81:108363. doi:10.1016/j.jnutbio.2020.108363 32388250

[59] Qiu K, Zhang X, Jiao N, Xu D, Huang C, Wang Y, Yin J. 2018. Dietary protein level affects nutrient digestibility and ileal microbiota structure in growing pigs. Anim Sci J 89:537–546. doi:10.1111/asj.12946 29271556

[60] Hamer HM, Jonkers D, Venema K, Vanhoutvin S, Troost FJ, Brummer R-J. 2008. Review article: the role of butyrate on colonic function. Aliment Pharmacol Ther 27:104–119. doi:10.1111/j.1365-2036.2007.03562.x 17973645

[61] Zhang M, Wang Y, Zhao X, Liu C, Wang B, Zhou J. 2021. Mechanistic basis and preliminary practice of butyric acid and butyrate sodium to mitigate gut inflammatory diseases: a comprehensive review. Nutr Res 95:1–18. doi:10.1016/j.nutres.2021.08.007 34757305

[62] Bischoff SC, Barbara G, Buurman W, Ockhuizen T, Schulzke J-D, Serino M, Tilg H, Watson A, Wells JM. 2014. Intestinal permeability – a new target for disease prevention and therapy. BMC Gastroenterol 14:189. doi:10.1186/s12876-014-0189-7 25407511 PMC4253991

[63] Sun Y, Zhang S, Nie Q, He H, Tan H, Geng F, Ji H, Hu J, Nie S. 2023. Gut firmicutes: relationship with dietary fiber and role in host homeostasis. Crit Rev Food Sci Nutr 63:12073–12088. doi:10.1080/10408398.2022.2098249 35822206

[64] Chu J, Wang Y, Zhao B, Zhang X-M, Liu K, Mao L, Kalamiyets E. 2019. Isolation and identification of new antibacterial compounds from Bacillus pumilus. Appl Microbiol Biotechnol 103:8375–8381. doi:10.1007/s00253-019-10083-y 31444521

[65] Yan H, Yun J, Ai D, Zhang W, Bai J, Guo J. 2018. Two novel cationic antifungal peptides isolated from Bacillus pumilus HN-10 and their inhibitory activity against Trichothecium roseum. World J Microbiol Biotechnol 34:21. doi:10.1007/s11274-017-2392-5 29302801

[66] Hou M, Lu Y, Ye M, Li N, Sun Y, Yao G, Wang J, Yin F, Peng Q, Jia S, Shi R, Wang X. 2025. Characterization and assessment of sheep-origin probiotic Bacillus licheniformis B63 strain for potential use in intestinal health and disease. Probiotics Antimicrob Proteins 17:781–793. doi:10.1007/s12602-023-10172-5 37874497

[67] Zhang N, Wang L, Wei Y. 2021. Effects of Bacillus pumilus on growth performance, immunological indicators and gut microbiota of mice. J Anim Physiol Anim Nutr (Berl) 105:797–805. doi:10.1111/jpn.13505 33675272

[68] Grant A, Gay CG, Lillehoj HS. 2018. Bacillus spp. as direct-fed microbial antibiotic alternatives to enhance growth, immunity, and gut health in poultry. Avian Pathol 47:339–351. doi:10.1080/03079457.2018.1464117 29635926

[69] Rhayat L, Maresca M, Nicoletti C, Perrier J, Brinch KS, Christian S, Devillard E, Eckhardt E. 2019. Effect of Bacillus subtilis strains on intestinal barrier function and inflammatory response. Front Immunol 10:564. doi:10.3389/fimmu.2019.00564 30984172 PMC6449611

[70] Truong Thy HT, Tri NN, Quy OM, Fotedar R, Kannika K, Unajak S, Areechon N. 2017. Effects of the dietary supplementation of mixed probiotic spores of Bacillus amyloliquefaciens 54A, and Bacillus pumilus 47B on growth, innate immunity and stress responses of striped catfish (Pangasianodon hypophthalmus). Fish Shellfish Immunol 60:391–399. doi:10.1016/j.fsi.2016.11.016 27836719

[71] Cai J, Wang N, Chen J, Wu A, Nepovimova E, Valis M, Long M, Wu W, Kuca K. 2022. Bacillus velezensis A2 inhibited the cecal inflammation induced by zearalenone by regulating intestinal flora and short-chain fatty acids. Front Nutr 9:806115. doi:10.3389/fnut.2022.806115 35360686 PMC8963806

[72] Xu L, Zhou Y, Zhan Z, Zhang W, Fu D, Zhao R, Chen X. 2022. Research Note: Effects of Bacillus coagulans X26 on the production performance, intestinal structure, short-chain fatty acids and flora composition of laying hens during the peak laying period. Poult Sci 101:101835. doi:10.1016/j.psj.2022.101835 35398755 PMC9006320

[73] Yan M, Yin W, Fang X, Guo J, Shi H. 2016. Characteristics of a water-forming NADH oxidase from Methanobrevibacter smithii, an archaeon in the human gut. Biosci Rep 36:e00410. doi:10.1042/BSR20160357 27737924 PMC5293585

[74] Shin NR, Whon TW, Bae JW. 2015. Proteobacteria: microbial signature of dysbiosis in gut microbiota. Trends Biotechnol 33:496–503. doi:10.1016/j.tibtech.2015.06.011 26210164

[75] Weiss GA, Hennet T. 2017. Mechanisms and consequences of intestinal dysbiosis. Cell Mol Life Sci 74:2959–2977. doi:10.1007/s00018-017-2509-x 28352996 PMC11107543

[76] Liu QX, Zhou Y, Li XM, Ma DD, Xing S, Feng JH, Zhang MH. 2020. Ammonia induce lung tissue injury in broilers by activating NLRP3 inflammasome via Escherichia/Shigella. Poult Sci 99:3402–3410. doi:10.1016/j.psj.2020.03.019 32616234 PMC7597683

[77] Meijer AJ. 2003. Amino acids as regulators and components of nonproteinogenic pathways. J Nutr 133:2057S–2062S. doi:10.1093/jn/133.6.2057S 12771365

[78] Fritz JH. 2013. Arginine cools the inflamed gut. Infect Immun 81:3500–3502. doi:10.1128/IAI.00789-13 23897606 PMC3811762

[79] Ruan D, Fouad AM, Fan QL, Huo XH, Kuang ZX, Wang H, Guo CY, Deng YF, Zhang C, Zhang JH, Jiang SQ. 2020. Dietary L-arginine supplementation enhances growth performance, intestinal antioxidative capacity, immunity and modulates gut microbiota in yellow-feathered chickens. Poult Sci 99:6935–6945. doi:10.1016/j.psj.2020.09.042 33248609 PMC7705054

[80] Zhang B, Lv Z, Li Z, Wang W, Li G, Guo Y. 2018. Dietary L-arginine supplementation alleviates the intestinal injury and modulates the gut microbiota in broiler chickens challenged by Clostridium perfringens. Front Microbiol 9:1716. doi:10.3389/fmicb.2018.01716 30108569 PMC6080643

[81] Jabecka A, Ast J, Bogdaski P, Drozdowski M, Pawlak-Lemaska K, Cielewicz AR, Pupek-Musialik D. 2012. Oral L-arginine supplementation in patients with mild arterial hypertension and its effect on plasma level of asymmetric dimethylarginine, L-citruline, L-arginine and antioxidant status. Eur Rev Med Pharmacol Sci 16:1665–1674. 23161038

[82] Kamzolova SV, Samoilenko VA, Lunina JN, Morgunov IG. 2023. Large-scale production of isocitric acid using Yarrowia lipolytica yeast with further down-stream purification. BioTech (Basel) 12:22. doi:10.3390/biotech12010022 36975312 PMC10046092

[83] Thurm C, Schraven B, Kahlfuss S. 2021. ABC transporters in T cell-mediated physiological and pathological immune responses. Int J Mol Sci 22:9186. doi:10.3390/ijms22179186 34502100 PMC8431589

[84] Durmus S, van der Valk M, Teunissen SF, Song JY, Wagenaar E, Beijnen JH, Schinkel AH. 2019. ABC transporters Mdr1a/1b, Bcrp1, Mrp2 and Mrp3 determine the sensitivity to PhIP/DSS-induced colon carcinogenesis and inflammation. Arch Toxicol 93:775–790. doi:10.1007/s00204-019-02394-w 30701287

[85] Han Y, Chen T, Li Y, Chen L, Wei L, Xiao L. 2019. Single-particle enumeration-based sensitive glutathione S-transferase assay with fluorescent conjugated polymer nanoparticle. Anal Chem 91:11146–11153. doi:10.1021/acs.analchem.9b01849 31402640

[86] Li T, Zhao X-P, Wang L-Y, Gao S, Zhao J, Fan Y-C, Wang K. 2013. Glutathione S-transferase P1 correlated with oxidative stress in hepatocellular carcinoma. Int J Med Sci 10:683–690. doi:10.7150/ijms.5947 23569432 PMC3619117

[87] Cheng Y-C, Lee H-L, Hwang Y, Kim SW. 2023. The effects of standardized ileal digestible His to Lys ratio on growth performance, intestinal health, and mobilization of histidine-containing proteins in pigs at 7 to 11 kg body weight. J Anim Sci 101:skac396. doi:10.1093/jas/skac396 36440959 PMC9838802

[88] Ribeiro LE, Albuini FM, Castro AG, Campos VJ, de Souza GB, Mendonça JGP, Rosa CA, Mendes TAO, Santana MF, da Silveira WB, Fietto LG. 2021. Influence of glucose on xylose metabolization by Spathaspora passalidarum. Fungal Genet Biol 157:103624. doi:10.1016/j.fgb.2021.103624 34536506

[89] Zeyue YU, Liyu H, Zongyuan LI, Jianhui S, Hongying C, Hairu H, Xiaoqin LI, Zhongchao S, Hongmei LI. 2022. Correlation between slow transit constipation and spleen deficiency, and gut microbiota: a pilot study. J Tradit Chin Med 42:353–363. doi:10.19852/j.cnki.jtcm.20220408.002 35610004 PMC9924678

[90] Gonçalves P, Martel F. 2013. Butyrate and colorectal cancer: the role of butyrate transport. Curr Drug Metab 14:994–1008. doi:10.2174/1389200211314090006 24160296

[91] Zhong X, Zhang Z, Wang S, Cao L, Zhou L, Sun A, Zhong Z, Nabben M. 2018. Microbial-driven butyrate regulates jejunal homeostasis in piglets during the weaning stage. Front Microbiol 9:3335. doi:10.3389/fmicb.2018.03335 30713531 PMC6345722

[92] Chapman MAS, Grahn MF, Hutton M, Williams NS. 1995. Butyrate metabolism in the terminal ileal mucosa of patients with ulcerative colitis. Br J Surg 82:36–38. doi:10.1002/bjs.1800820115 7881952

[93] Nugraha RYB, Jeelani G, Nozaki T. 2022. Physiological roles and metabolism of γ-aminobutyric acid (GABA) in parasitic protozoa. Trends Parasitol 38:462–477. doi:10.1016/j.pt.2022.02.004 35264298

[94] Kim M, Oh S, Kim S, Ji M, Choi B, Bae J-W, Lee YS, Paik M-J, Lee S. 2023. Alcohol perturbed locomotor behavior, metabolism, and pharmacokinetics of gamma-hydroxybutyric acid in rats. Biomed Pharmacother 164:114992. doi:10.1016/j.biopha.2023.114992 37301134

[95] Mamelak M. 2025. Depression and the glutamate/GABA-glutamine cycle. Curr Neuropharmacol 23:75–84. doi:10.2174/1570159X22666240815120244 PMC11519819 39150032

[96] Marri L, Jansson AM, Christensen CE, Hindsgaul O. 2017. An enzyme-linked immunosorbent assay for the detection of diacetyl (2,3-butanedione). Anal Biochem 535:12–18. doi:10.1016/j.ab.2017.07.021 28739133

[97] Anders MW. 2017. Diacetyl and related flavorant α-Diketones: biotransformation, cellular interactions, and respiratory-tract toxicity. Toxicology 388:21–29. doi:10.1016/j.tox.2017.02.002 28179188

[98] Laroute V, Tormo H, Couderc C, Mercier-Bonin M, Le Bourgeois P, Cocaign-Bousquet M, Daveran-Mingot M-L. 2017. From genome to phenotype: an integrative approach to evaluate the biodiversity of Lactococcus lactis. Microorganisms 5:27. doi:10.3390/microorganisms5020027 28534821 PMC5488098

[99] Morris JB, Hubbs AF. 2009. Inhalation dosimetry of diacetyl and butyric acid, two components of butter flavoring vapors. Toxicol Sci 108:173–183. doi:10.1093/toxsci/kfn222 18940962 PMC2644402

