# Supplementary material for: Integrated metabolome and microbiome analysis deciphers the effects of resveratrol and β-hydroxy-β-methylbutyric acid on jejunal function under different protein levelshttps://webofscience.clarivate.cn/wos/woscc/full-record/WOS:000624324600014in Tibetan sheep
Source: Microbiol Spectr. 2026 Jan 21;14(3):e02843-25. doi: 10.1128/spectrum.02843-25 (PMC12955390; doi:10.1128/spectrum.02843-25)
Supplement: Table S1 — Jejunal primer sequences. [file spectrum.02843-25-s0001.docx]

TableS1 Primers used in qRT-PCR

| Name | Primer sequence (5’-3’) | Tm (℃) | Product length |
| --- | --- | --- | --- |
| claudin-1 | F-CCTGCTGTGCTGCTCCTGTC  R-GAAGGTGCTGGCTTGGGATAGG | 61.6  61.4 | 75bp |
| occludin | F-CGAGAAGCGACCGTATCCAGAG  R-TCCAAGTTACCACTGCTGCTGTAG | 61.4  59.6 | 129bp |
| muc-2 | F-ACGACTCCTACGCCCTCCTG  R-ACGCTGCCATCCGACTTGAAG | 61.6  59.5 | 130bp |
| ZO-1 | F-GGGCAAGTTAAAGATGGTGGTTCAG  R-GAGGCGTCAGCAGAGTGGATG | 59.6  61.5 | 93bp |
| TNF-α | F-ACGGCGTGGAGCTGAAAGAC  R-CTGAAGAGGACCTGCGAGTAGATG | 59.5  61.3 | 79bp |
| IL-6 | F-TCTAATAACCACTCCAGCCACACAC  R-TTGCGTTCTTTACCCACTCGTTTG | 59.6  57.9 | 77bp |
| IL-1β | F-GGCAGGCAGTGTCGGTCATC  R-CCTCAGGTCATCATCACGGAAGAC | 61.6  61.3 | 83bp |
| IL-10 | F-AATGAAGGACCAACTGAACAGCATG  R-TCCGACAAGGCTTGGCAACC | 57.9  59.5 | 87bp |
| β-Actin | F-AGCAAGCGTGGCATCCTAACC  R-ATCTTCTCCATGTCGTCCCAGTTG | 59.5  59.6 | 77bp |
